# Probiotics as Modulators of Adult Neurogenesis and Synaptic Plasticity: New Perspectives in the Pathophysiology and Treatment of Affective Disorders

**DOI:** 10.3390/biomedicines14030637

**Published:** 2026-03-12

**Authors:** Gilberto Uriel Rosas-Sánchez, León Jesús Germán-Ponciano, María Isabel Pérez-Vega, Oscar Gutiérrez-Coronado, José Luis Muñoz-Carrillo, Alejandro David Soriano-Hernández, Abril Alondra Barrientos-Bonilla, Carmen Gabriela Rosales-Muñoz, Cesar Soria-Fregozo

**Affiliations:** 1 Programa de Estancias Posdoctorales por México, Secretaría de Ciencia, Humanidades, Tecnología e Innovación SECIHTI, Centro Universitario de Los Lagos, Universidad de Guadalajara, Lagos de Moreno 47460, Jalisco, Mexico; mcbjlmc@gmail.com (J.L.M.-C.); alejandro.soriano@lagos.udg.mx (A.D.S.-H.); 2 Departamento de Ciencias de la Tierra y de la Vida, Centro Universitario de Los Lagos, Universidad de Guadalajara, Lagos de Moreno 47460, Jalisco, Mexico; misabel.perez@academicos.udg.mx (M.I.P.-V.); carmen.rosales9819@academicos.udg.mx (C.G.R.-M.); 3 Laboratorio de Neurofarmacología, Instituto de Neuroetología, Universidad Veracruzana, Xalapa 91190, Veracruz, Mexico; lgerman@uv.mx; 4 Laboratorio de Inmunología, Centro Universitario de los Lagos, Universidad de Guadalajara, Lagos de Moreno 47460, Jalisco, Mexico; oscar.gcoronado@academicos.udg.mx; 5 Coordinación Universitaria para la Sustentabilidad (Cosustenta), Universidad Veracruzana, Xalapa 91000, Veracruz, Mexico; abribarrientos@uv.mx

**Keywords:** probiotics, neurogenesis, neuroplasticity, MGB axis, anxiety, depression

## Abstract

Affective disorders, such as major depressive disorder and anxiety disorders, represent a major global health burden, with current treatments proving inadequate for a substantial proportion of patients. Emerging research highlights the microbiota–gut–brain (MGB) axis as a crucial bidirectional communication system influencing brain function and neuroplasticity through neural, endocrine, immune, and metabolic pathways. This narrative review examines probiotics—live beneficial microorganisms—as modulators of adult neurogenesis and synaptic plasticity, two processes fundamentally implicated in the pathophysiology of affective disorders. Preclinical evidence demonstrates that specific strains, particularly from the *Lactobacillus* and *Bifidobacterium* genera, promote hippocampal neurogenesis and synaptic function through epigenetic regulation via short-chain fatty acids (SCFAs), notably butyrate-mediated histone deacetylase inhibition, modulation of neuroinflammatory pathways, regulation of neurotransmitter receptor expression across glutamatergic, GABAergic, and monoaminergic systems, and production of neuroactive peptides. Clinical evidence from randomized controlled trials and recent meta-analyses indicates that probiotic supplementation produces significant reductions in depressive and anxiety symptoms, with effects correlating to changes in gut microbiota composition and peripheral neuroplasticity biomarkers, particularly brain-derived neurotrophic factor (BDNF). However, significant methodological limitations persist, including small sample sizes, lack of standardization in probiotic strains and dosages, inconsistent outcome measures, and considerable interindividual variability. While the mechanistic and clinical evidence is biologically plausible and directionally promising, it is not yet sufficient to support definitive therapeutic recommendations. Future research must prioritize adequately powered clinical trials with standardized consortia, comprehensive multi-omics biomarker panels, and precision psychobiotic strategies guided by microbiome-defined patient stratification.

## 1. Introduction

Affective disorders, such as major depression and anxiety disorders, represent a significant global health burden, characterized by debilitating emotional and cognitive dysfunction. According to the World Health Organization, in 2021 approximately 1.1 billion people worldwide—nearly one in every seven—were living with a mental disorder, with anxiety and depressive disorders being the most prevalent, affecting 359 million and 280 million individuals, respectively [1]. Depression is projected to become the leading cause of global disease burden by 2030, and mental health conditions are the second-most common cause of long-term disability worldwide [1]. Despite this staggering burden, fewer than one in four individuals with anxiety disorders receive adequate treatment, reflecting a critical gap between need and access to effective interventions. Despite advances in pharmacological and psychological interventions, a substantial portion of patients experience inadequate responses, highlighting the urgent need for novel therapeutic strategies and a deeper understanding of their underlying pathophysiology [2]. Recent scientific research has increasingly focused on the intricate interplay between the gut microbiome and the brain, a bidirectional communication system known as the “MGB axis,” as a critical determinant of mental health and disease [3,4,5]. This axis involves a complex network of neural, endocrine, and immune pathways through which gut microbiota can profoundly influence brain function, including mood, cognition, and emotional regulation [3,6,7].

Neuroplasticity, the remarkable ability of the nervous system to adapt and reorganize itself throughout life, is central to brain function. Two fundamental forms of neuroplasticity—adult neurogenesis and synaptic plasticity—are especially relevant to the etiology and treatment of affective disorders. Adult neurogenesis is the ongoing generation of new neurons in specific regions of the adult brain, notably the subgranular zone of the hippocampal dentate gyrus [8,9]. These new neurons play crucial roles in learning, memory, and emotional regulation, and their dysfunction is strongly implicated in stress-related neuropsychiatric conditions such as anxiety and depression [10,11]. Synaptic plasticity involves long-term changes in the strength and efficacy of synaptic connections between neurons, enabling the brain to learn from past experiences and form lasting memories [2]. Disrupted synaptic plasticity is considered a fundamental mechanism underlying a wide range of neuropsychiatric disorders, including depression, schizophrenia, and addiction [2]. Changes in these neuroplastic processes can contribute to the development and persistence of depressive behavioral states [12], with antidepressants often acting by reversing stress-induced effects on synaptic function and enhancing plasticity [13].

The burgeoning field of microbiome research has opened new avenues for understanding and treating affective disorders, identifying the gut microbiota as a potent modulator of neurogenesis and synaptic plasticity [14,15]. Probiotics, defined as living microorganisms that, when administered in adequate amounts, confer a health benefit to the host, are emerging as promising agents in this context [16]. By interacting with gut microbial communities and alleviating gut dysbiosis, probiotics can modulate the MGB axis and potentially influence cognitive function and emotional stability [17]. Studies have shown that probiotic supplementation can positively affect anxiety and depressive symptoms in humans, suggesting their potential to improve mental well-being [18,19]. It is important to note, however, that probiotic effects are highly strain-specific: different strains, even within the same genus or species, can exert entirely distinct—and sometimes opposing—effects on gut microbiota composition, neurotransmitter receptor expression, and neuroplasticity-related pathways, precluding broad generalizations across probiotic preparations [20,21].

This narrative review comprehensively examines the role of probiotics as modulators of adult neurogenesis and synaptic plasticity, offering new perspectives on the pathophysiology and treatment of affective disorders. Adopting a dual mechanistic-clinical scope, it systematically synthesizes preclinical evidence from rodent models, germ-free (GF) preparations, and in vitro systems, alongside clinical evidence from randomized controlled trials and translational biomarker studies in human populations. Throughout, both levels of evidence are explicitly distinguished to delineate the current boundaries of translational knowledge and to avoid unwarranted extrapolation from animal to human contexts. Key mechanistic pathways addressed include epigenetic regulation via SCFAs, modulation of inflammatory pathways, neurotransmitter receptor expression, and neuroactive peptide signaling, while clinical sections evaluate their correspondence with measurable outcomes in mood disorders and brain plasticity biomarkers.

Despite the growing mechanistic evidence linking the MGB axis to neuroplasticity, several critical knowledge gaps persist. First, the precise probiotic strains and minimum effective doses required to produce measurable neurogenic and synaptic effects in humans remain undefined. Second, whether probiotic-induced changes in BDNF, SCFA profiles, and inflammatory markers translate into clinically meaningful neuroplastic outcomes—beyond symptomatic improvement—has not been established through mechanism-specific human trials. Third, the optimal intervention windows across the lifespan, the persistence of effects following discontinuation, and the extent to which interindividual microbiota composition determines therapeutic response are largely unresolved. This review aims to systematically address these gaps by integrating preclinical and emerging clinical evidence.

## 2. Search Strategy

### 2.1. Design

This study was designed as a narrative literature review on the effects of probiotics as modulators of adult neurogenesis and synaptic plasticity in affective disorders, analyzing information from scientific papers and book chapters to provide an informative, critical, and useful synthesis of the topic. In a narrative review, there are no predetermined research questions or specific search strategy, only a topic of interest. Recognizing that narrative reviews lack systematic methods for identifying, evaluating, and synthesizing information, which could lead authors to include or exclude information to support a particular position [22,23], we describe the parameters used to include or exclude studies to ensure the objective inclusion of information. These included conducting a search and identifying keywords, reviewing abstracts and articles, and documenting results [24].

To ensure epistemological clarity, evidence in this review is categorized by origin: (i) preclinical evidence, including in vitro studies, GF animal models, and rodent behavioral paradigms, which provides mechanistic resolution but limited translational fidelity; and (ii) clinical evidence, consisting of randomized controlled trials, meta-analyses, and translational biomarker studies in human populations, which offers direct therapeutic relevance but currently limited mechanistic specificity. When evidence is exclusively from preclinical sources, this is explicitly noted to avoid overstating clinical applicability.

### 2.2. Criteria

The inclusion criteria focused on research articles, reviews, and statements in which the effects of various probiotic strains were investigated in preclinical and clinical studies during chronic treatment interventions lasting four weeks or longer, consistent with the minimum period reported in the literature as necessary for probiotics to exert measurable effects on gut microbiota composition and host physiology, as well as their mechanisms of action on adult neurogenesis and synaptic plasticity, in English- and Spanish-language articles. Only studies involving chronic probiotic treatment were included, as the beneficial effects of probiotics require sufficient time to consolidate and become clinically measurable. This requirement reflects the biological reality that probiotics must first colonize the gut, progressively modulate the existing microbial community, and subsequently exert their immunomodulatory and metabolic effects on the host—a cascade of processes that cannot be adequately captured through acute or short-term administration protocols. Furthermore, most conditions for which probiotics are studied (e.g., irritable bowel syndrome, inflammatory bowel disease, improvement in immune function, neuroplasticity, emotional and affective disorders) are chronic and require long-term intervention. Exclusion criteria were studies without full-text access, unofficial websites, duplicate publications, and doctoral dissertations.

### 2.3. Article Research

Data on the topic described in the inclusion criteria were searched in specialized databases such as PubMed, ScienceDirect, Web of Science, and Scopus, using the following Boolean operators and combinations of terms in each database: “probiotics,” “anxiety,” “depression,” “neurogenesis,” “MGB axis,” “bacterial metabolites,” “BDNF,” “neuroplasticity,” “brain function,” “treatment,” “animal,” “models,” “clinical trials,” and “probiotic mechanism of action,” with a language restriction to English and Spanish only.

## 3. Neurobiological Foundations of Affective Disorders

Affective disorders, such as major depressive disorder and anxiety disorders, are characterized by persistent disturbances in mood, emotion, and cognition, representing a significant global health challenge. Understanding the neurobiological underpinnings of these disorders is crucial for developing more effective treatments. Research over the past decades has increasingly focused on alterations in fundamental brain plasticity mechanisms, namely adult neurogenesis and synaptic plasticity, as key contributors to their pathophysiology [25].

### 3.1. Alterations in Adult Neurogenesis in Depression and Anxiety

Adult neurogenesis, the process of generating new neurons in the adult brain, primarily occurs in the subgranular zone of the hippocampal dentate gyrus. These newly born neurons are critical for functions such as learning, memory, and emotional regulation [9]. Evidence from both human and animal studies suggests that dysregulation of adult hippocampal neurogenesis (AHN) is strongly linked to stress-related neuropsychiatric conditions, including anxiety and depression [8,10].

In major depressive disorder (MDD), the “neurogenic hypothesis of depression” proposes that impairments in AHN contribute to the etiology of the disorder, and that the therapeutic effects of antidepressants are mediated, at least in part, by an increase in neurogenesis [26,27]. Chronic stress, a significant risk factor for depression and anxiety, can lead to a transient reduction in neurogenesis in animal models, resulting in an anxiety- and depressive-like phenotype [28]. This reduction in AHN is thought to impair the normal function of the dentate gyrus (DG), affecting processes like pattern separation and cognitive flexibility, which can contribute to depressive and anxiety-like behaviors [8,29]. Conversely, activating newborn neurons has been shown to suppress depression- and anxiety-like behaviors in experimental models, supporting the causal link between neurogenesis and mood [26].

The hippocampus plays a critical role in mood regulation, partly due to its connections with emotion-related brain regions such as the amygdala and anterior cingulate cortex, and its feedback role in regulating the hypothalamic–pituitary–adrenal (HPA) axis [27]. Dysregulation of neural circuits involving the hippocampus, amygdala, and medial prefrontal cortex (mPFC) has been demonstrated in patients with anxiety-related disorders, affecting learning, memory, and emotional processing [30]. Structural Magnetic Resonance Imaging (MRI) studies consistently report hippocampal volume reduction in MDD patients, suggesting structural changes indicative of impaired neurogenesis or neuronal atrophy, although findings in anxiety disorders are less consistent [31,32].

However, it should be noted that the existence and functional significance of adult hippocampal neurogenesis in humans remains a subject of active scientific controversy. While robust neurogenic activity has been consistently demonstrated in rodent models, postmortem human studies have yielded conflicting findings, with some reporting thousands of immature neurons in the adult dentate gyrus and others detecting negligible neurogenesis beyond early childhood. This discrepancy is attributable to methodological differences in tissue fixation, immunohistochemical markers, and postmortem interval [9]. This ongoing debate does not invalidate the neurogenic hypothesis of depression, which retains strong preclinical support, but it does underscore the importance of interpreting probiotic-induced neurogenic effects—currently established primarily in animal models—with appropriate caution regarding their direct translation to human pathophysiology and therapeutic outcomes.

### 3.2. Synaptic Plasticity Deficits in Experimental Models and Clinical Findings

Synaptic plasticity refers to the ability of synapses, the junctions between neurons, to strengthen or weaken over time in response to activity. This fundamental process underlies learning, memory, and adaptive behavioral responses. Dysfunctional plasticity is associated with a wide spectrum of neuropsychiatric disorders, including depression, schizophrenia, addiction, and posttraumatic stress disorder [2]. Experimental models of chronic stress and depressive-like behaviors consistently demonstrate impairments in neuroplasticity, such as neuronal atrophy and synaptic loss in the mPFC and hippocampus [33]. These structural and functional changes in synaptic connections are believed to contribute to the cognitive and emotional symptoms of affective disorders [34].

The understanding of depression has shifted from a focus on monoamine deficiency to a “neuroplasticity hypothesis.” This hypothesis suggests that impairments in neuroplasticity are central to the disease and that the efficacy of antidepressant treatments involves restoring or enhancing these plastic processes [27]. This includes long-term activity-dependent changes in synaptic strength (“plasticity”) and shifts in the set points for the ease of induction of future long-term changes (“metaplasticity”), which may be critical to establishing and reversing a depressive behavioral state [12]. Altered connectivity and network function have been observed in the brains of depressed patients, affecting excitatory glutamate neurons and inhibitory GABA interneurons [34]. Furthermore, neuromodulation of synaptic plasticity and network coupling in the hippocampal–prefrontal pathway is implicated in behavioral and circuit alterations related to neuropsychiatric conditions [35].

### 3.3. Functional Implications of These Alterations in Emotional Processing

Alterations in adult neurogenesis and synaptic plasticity have profound functional implications for emotional processing in individuals with affective disorders. The hippocampus plays a critical role in modulating emotional behavior and stress resilience, particularly in its ventral region [8]. Deficits in AHN can impair hippocampal function, leading to difficulties in processes such as pattern separation and cognitive flexibility, which are known to be affected in depression and anxiety and may contribute to their etiology [8].

Dysregulated neuroplasticity in key brain regions contributes to maladaptive emotional responses. Impaired synaptic plasticity can result in an inability to appropriately extinguish fear responses, contributing to anxiety disorders, or in a reduced capacity for reward learning and emotional resilience in depression. The prefrontal cortex, amygdala, and hippocampus are central to emotional regulation and memory, and disruptions within their neural circuits contribute to the maladaptive behaviors and emotional dysregulation observed in these disorders [30,36]. The functional implications extend to how the brain transitions from a healthy emotional state to one marked by anxiety or depression, with interactions between these structures modulated by neurotransmitters such as serotonin, dopamine, and norepinephrine [36]. Changes in behavior and hippocampal-dependent circuits, attributed to abnormalities at the molecular, structural, and synaptic levels, impact how individuals process and respond to emotional stimuli [27].

### 3.4. Biomarkers of Neuroplasticity in Affective Disorders

The identification of robust, reliable, and valid biomarkers for psychiatric disorders is essential for objective diagnosis, personalized treatment recommendations, and monitoring therapeutic efficacy [37]. While direct measures of neurogenesis and synaptic plasticity in living humans remain challenging, various indirect biomarkers are being explored. Studies of neuroimaging markers consistently report hippocampal volume reduction in patients with MDD, and sometimes in anxiety disorders. For example, individuals with depression and comorbid anxiety show significantly higher amygdala volumes, while comorbid anxiety reduces the effects of depression on brain structure [32]. These structural changes are often considered indicative of impaired neurogenesis or neuronal atrophy [31].

Studies using functional magnetic resonance imaging (fMRI) have demonstrated alterations in amygdala–prefrontal connectivity, both structural and functional, in anxiety and depression. These common connectivity deficits indicate reduced functional coupling in corticolimbic circuitry, a system critical for emotion regulation [31]. Neuroimaging findings also suggest unique neuroanatomical profiles for these disorders [31]. Personalized brain circuit scores, derived from fMRI, are being developed to quantify neurobiological dysfunctions and identify clinically distinct biotypes in depression and anxiety, moving toward precision medicine in psychiatry [38]. Advances in neuroimaging and neurostimulation techniques, including eigenvector centrality mapping and machine learning-driven analyses, show promise for individualized care [36].

Molecular and peripheral assays have shown that neurotransmitters such as serotonin (5-HT), dopamine (DA), and norepinephrine (NE) play critical roles in mood regulation, stress response, and neuroplasticity. Dysregulation in these systems is implicated in the pathophysiology of affective disorders [36]. Genetic factors influencing these conditions are also being investigated to provide a more comprehensive understanding [36]. Psychiatry is actively seeking robust and reliable biomarkers, reviewing evidence for promising candidates across various neuroimaging, genetic, molecular, and peripheral assays to determine susceptibility, presence of illness, and to predict treatment response or safety [37]. These biomarkers offer insights into the neurobiological state of affective disorders, providing avenues for more precise diagnosis and personalized treatment approaches targeting neuroplasticity. Continued research into neuroplasticity is vital for the evolution of therapeutic strategies [39].

To provide a structured overview of the biomarker landscape discussed in this section, Table 1 summarizes the principal neuroplasticity biomarkers reported in studies of affective disorders and probiotic interventions, organized by category—including neuroimaging, neurotrophic, neurotransmitter, inflammatory, metabolic, microbiota-compositional, epigenetic, and electrophysiological markers. For each category, the table specifies the measurement approach used, the characteristic findings observed in affective disorders, and the evidence linking each biomarker to probiotic intervention outcomes. This classification underscores the fundamentally indirect nature of the neuroplasticity biomarkers currently available in clinical research—none of which are direct measures of neurogenesis or synaptic plasticity in the living human brain—and highlights the varying levels of translational evidence supporting each biomarker class, from well-established peripheral surrogates such as serum BDNF and inflammatory indices to emerging metabolomic markers such as circulating SCFA profiles. Collectively, these biomarkers provide the empirical foundation for the clinical sections of this review, and their systematic integration into future probiotic trials will be essential for bridging the mechanistic evidence from preclinical models with measurable neuroplastic outcomes in human populations.

## 4. The Gut–Microbiota–Brain Axis: Mechanisms of Bidirectional Communication

The study of the enteric nervous system (ENS) gained relevance following the work of Michael Gershon in the 1990s, which described its functional autonomy and role in gastrointestinal regulation [66,67]. However, the concept of the MGB axis did not emerge as a scientific paradigm until the early 21st century. This axis integrates neural, endocrine, immunological, and metabolic signals [67,68], highlighting the role of the gut microbiota in regulating anxiety, mood, cognition [69,70,71], immunomodulation [72,73], energy metabolism [74], and disease prevention, including neurodegenerative diseases [71,75].

Let us discuss the elements of this axis, starting with the gut microbiota. Approximately 100 trillion microorganisms—mostly bacteria, but also viruses, fungi, and protozoa—inhabit the lumen of the human gastrointestinal tract. The microbiota is considered a virtual organ that evolves with its host from gestation to death. During gestation, the maternal microbiota, dietary patterns, stress, and antibiotic use directly influence the microbial composition of the newborn, as bacterial colonization can begin in utero via the transplacental route. The most abundant genera are *Firmicutes*, *Tenericutes*, *Proteobacteria*, *Bacteroidetes*, and *Fusobacteria* [76]. Subsequently, the type of delivery is a determining factor. Vaginal delivery exposes the newborn to beneficial bacteria, mainly from the genera *Lactobacillus* and *Bifidobacterium*, while cesarean section is associated with initial colonization characterized by less diversity and a greater presence of skin, environmental, and hospital-acquired bacteria. This can predispose individuals to immunological, metabolic, and neurodevelopmental disorders [77,78]. After birth, breastfeeding promotes eubiosis and supports immunoneurological maturation. In contrast, formula feeding tends to increase *Firmicutes* and *Bacteroidetes* species with lower immunomodulatory and metabolic capacity. During the complementary feeding stage, the introduction of fiber, fruits, and vegetables increases the diversity of the gut microbiota and the production of neuroactive metabolites [79,80]. It should provide a concise and precise description of the experimental results, their interpretation, and the experimental conclusions that can be drawn.

It has been observed that changes in initial microbial colonization can affect myelination, maturation of the blood–brain barrier (BBB), neuronal generation and migration, as well as the production of brain trophic factors. These changes are associated with an increased risk of autism spectrum disorders (ASDs), attention deficit hyperactivity disorder (ADHD), anxiety, and cognitive or behavioral difficulties in childhood and adulthood [78,79,80,81]. In adulthood, lifestyle and diet determine the state of homeostasis or dysbiosis in the gut microbiota and its impact on overall health.

The microbiota is involved in the immune, metabolic, structural, and neurological systems [82,83,84]. Certain bacterial strains in the microbiota, such as *Akkermansia muciniphila*, have been shown to improve insulin sensitivity, while others, such as *Bifidobacterium longum*, *Bacteroides uniformis*, *Roseburia inulinivorans*, *Eubacterium rectale*, and *Faecalibacterium prausnitzii*, modulate the stress response and have a positive effect on maintaining mental health [85,86,87]. Furthermore, the microbiota is involved in the synthesis of essential vitamins (such as vitamin K and some B vitamins) and in the detoxification of xenobiotic compounds, directly impacting overall health [88].

The second component of this axis is the intestines, which play crucial roles in the digestion and absorption of nutrients. The human intestine, in both its small and large sections, is organized into four concentric layers: the mucosa, submucosa, muscularis propria, and serosa. Seven different intestinal cell lineages have been identified: enterocytes, responsible for absorption and barrier function; goblet cells, which produce mucus; Paneth cells, which secrete antimicrobial peptides and growth factors; microfold cells, which maintain and communicate with lymphoid follicles; cup cells; tuft cells, responsible for antigen presentation; and enteroendocrine cells (EECs), which release hormones and facilitate gut–brain communication. The mucosal layer serves as the primary interface for dynamic interaction with the microbiota and is composed mainly of enterocytes, mucus-secreting goblet cells, and EECs [89,90]. EECs are dispersed effector cells that directly detect a wide range of luminal ligands, from nutrients (glucose, lipids, amino acids) to microbial metabolites derived from fiber fermentation, such as SCFAs, and pathogen-associated molecular patterns (PAMPs). Furthermore, EECs are connected to the nervous system via neuropods that extend into the enteric nervous system and nearby structures, forming a bidirectional communication network. This interaction allows hormonal and neural signals to modulate functions with a profound influence on neuroinflammatory, behavioral, and metabolic states at a systemic level [89,91].

The final element of this axis, the brain, is directly related to the physical structures that enable communication from the gut to the central nervous system (CNS). Communication between the brain and the gut is organized through a hierarchical, bidirectional neural network. The primary pathway for sensory information from the gut to the brain is the vagus nerve. Signals travel along its afferent fibers, whose neuronal cell bodies are located in the ganglion nodosum, until they make their first central synapse in the nucleus of the solitary tract (NTS) in the brainstem. The NTS acts as a relay center, integrating visceral information and transmitting it to other key brain nuclei. These include the dorsal motor nucleus of the vagus nerve (DMV), which generates motor commands for return signals to the gut; the area postrema (which regulates the digestive process); the hypothalamus (a central regulator of appetite, stress, and metabolism); and the amygdala (involved in emotional responses). This connectivity allows the intestinal state to directly influence homeostasis, eating behavior, and affective state [92,93].

Vagal afferent pathways have a strategic distribution, located in the intestinal villi, underlying the mucosa, and surrounding glands, which facilitates the indirect detection of bacterial signals through interaction with endocrine cells. These cells can release 5-HT and peptides such as cholecystokinin in response to microbial stimuli, thereby activating specific receptors on vagal fibers. The involvement of molecules such as SCFAs, especially butyrate and oleate, has also been described; these can modulate vagal activity, influencing neurotransmission and systemic neuroendocrine responses [94].

Additionally, vagal activation has anti-inflammatory properties that regulate the intestine by activating the HPA axis and releasing cortisol, as well as through a vago-vagal reflex, which has an anti-tumor necrosis factor (TNF) effect called the cholinergic anti-inflammatory pathway. In this pathway, released acetylcholine can inhibit the synthesis of pro-inflammatory cytokines, thus regulating immune reactivity at both the intestinal and systemic levels [92,95].

The response from the brain to the gut follows the reverse pathway. Excitatory parasympathetic commands originate in neurons of the vagus nerve, travel along the efferent fibers of the vagus nerve, and activate the ENS, specifically at the level of the submucosal and myenteric plexuses. Meanwhile, inhibitory sympathetic commands, originating in the spinal cord and relayed in the prevertebral ganglia, negatively modulate these same functions [93].

In this circuit, EECs are the primary sensory elements that initiate the signaling pathway for the release of neurotransmitters (such as 5-HT) and hormones. These messengers directly activate neighboring vagal nerve terminals. Furthermore, as previously mentioned, EECs possess neuropods that can form a direct physical synapse (similar to a connection between two neurons) with the afferent fibers of the ENS and the vagus nerve [93,96].

Another communication mechanism of the MGB axis is the immune system. The gut is the body’s largest immune organ, with its mucosa containing more than 70–80% of all immune cells [66]. The gut microbiota functions as a “trainer” and modulator of the mucosal immune system. Beneficial bacteria, such as *Bifidobacterium* and *Lactobacillus*, support immune tolerance, the integrity of the epithelial barrier, and the regulated production of anti- and pro-inflammatory cytokines. However, when dysbiosis occurs, it favors the proliferation of pro-inflammatory bacteria, such as certain *clostridia* and *enterobacteria*, which can trigger excessive immune responses and contribute to chronic inflammation [97,98].

Microbiota-mediated immune activation begins with the recognition of PAMPs, such as lipopolysaccharide (LPS), which are detected by Toll-like receptors (TLRs) on innate immune cells and epithelial cells. The LPS-TLR interaction triggers intracellular signaling cascades that lead to the production and release of proinflammatory cytokines (IL-1β, IL-6, TNF-α, IFN-γ), promoting the activation of microglial cells and neuroinflammation, a central process in the development of neurodegenerative and affective disorders such as depression and anxiety [94,99].

The endocrine system is also a communication pathway of the MGB axis. EECs in the intestinal mucosa release multiple gastrointestinal hormones, such as ghrelin, insulin, peptide YY (PYY), and glucagon-like peptide-1 (GLP-1), in response to nutrients, nerve signals, and bacterial products [47,90,100]. Ghrelin and leptin, gastrointestinal hormones, regulate appetite and energy homeostasis, but also have neuropsychiatric effects, such as modulating motivation, stress, and food reward. Their levels have been shown to be altered by the composition and function of the gut microbiota [101].

The production of microbial metabolites is also recognized as a communication pathway of the MGB axis, with emphasis on the involvement of SCFAs, especially acetate, propionate, and butyrate. These compounds are generated mainly from the anaerobic fermentation of undigested dietary fiber by intestinal bacteria, primarily from the genera *Firmicutes* and *Bacteroidetes* [102]. Butyrate has epigenetic effects on brain gene expression, microglial maturation, and synaptic plasticity; it also increases dendritic spines and the formation of new neuronal connections. Additionally, it strengthens enterocyte tight junctions and the BBB, limiting the entry of proinflammatory cytokines and microbial toxins into the CNS [55,103,104,105]. Acetate and propionate cross the BBB and modulate hypothalamic circuits involved in energy homeostasis and regulation of the stress response. In animal models, SCFA supplementation produces anxiolytic and antidepressant effects, while deficiencies are associated with increased vulnerability to inflammation and chronic stress [55,56,57,58].

The gut microbiota also directly synthesizes neuromodulators essential for brain function. *Bifidobacterium* and *Lactobacillus* produce gamma-aminobutyric acid (GABA), a neurotransmitter involved in regulating the stress response, anxiety, and emotional tone. Other bacterial species, such as *Escherichia coli* and *Bacillus subtilis*, participate in the production of DA and NE, neurotransmitters central to motivation, cognition, and mood regulation. Although most of these neurotransmitters do not cross the BBB, they can exert effects locally in the ENS and modulate signals transmitted to the brain via sensory afferents and the vagal response [102,106,107,108,109]. 5-HT is another neurotransmitter highly produced in the gut; approximately 90% of the body’s 5-HT is synthesized in the ECS. Although gut-derived 5-HT does not cross the BBB, microbial production of precursors such as tryptophan, as well as modulation of the 5-HT transporter and reuptake receptor, can influence the amount of 5-HT available in the brain and, consequently, emotional and behavioral balance [48,100].

As we have seen, the gut microbiota has emerged as a fundamental player in human physiology, transcending its digestive role to become central to the bidirectional communication of the MGB axis. Maintaining a balanced gut microbiota is a key factor for mental and neurological health, opening new therapeutic possibilities based on dietary, probiotic, and psychobiotic interventions aimed at restoring healthy gut–brain communication.

## 5. Probiotics and Adult Neurogenesis

Adult neurogenesis, the process of generating new neurons in the adult brain, primarily occurs in the subgranular zone of the hippocampal DG and the subventricular zone [8]. This continuous production of new neurons in the adult brain affects cognitive functions, particularly memory, and also contributes to affective functions [9,110]. Dysregulation of adult AHN is strongly linked to stress-related neuropsychiatric conditions such as anxiety and depression, and deficits in this process may underlie the pathophysiology of affective disorders [8,10]. Emerging research highlights the significant influence of the gut microbiota on adult neurogenesis, with probiotics—live microorganisms that confer health benefits—serving as important modulators of this process through complex molecular mechanisms [51,111].

### 5.1. Preclinical Evidence on the Modulation of Hippocampal Neurogenesis by Specific Probiotics Subsection

Preclinical studies, primarily in rodent models, have provided substantial evidence that specific probiotic strains can modulate neurogenesis in the hippocampus. These studies often report increased cell proliferation, neuronal differentiation, and survival of new neurons in the dentate gyrus following probiotic administration. The MGB axis, a key communication channel between the intestinal microbiome and the brain, facilitates the microbial impact on neural functions [51].

For example, certain *Lactobacillus* and *Bifidobacterium* strains have been shown to promote adaptive cognitive functioning, mediated by pathways including AHN [51]. In models of chronic stress or gut dysbiosis, often induced by antibiotics, probiotics have been found to restore neurogenesis to basal levels or even enhance it. Specific *Bifidobacteria* strains contribute to the establishment of functional neural circuits during postnatal development by promoting synapse formation and microglial function [112]. Additionally, treatment with gut *Bifidobacteria* has been observed to improve hippocampal plasticity and cognitive behavior in healthy adult rats, consistent with enhanced neurogenesis [113]. The *Bifidobacterium* and *Lactobacillus* genera have shown the strongest positive correlation with neurogenesis and BDNF [114].

Germ-free (GF) animals, which lack a microbiota, exhibit significantly reduced AHN and altered neurodevelopmental trajectories [115,116]. These animals show defects in microglia, with altered cell proportions and an immature phenotype, indicating the gut microbiome’s role in microglial maturation [117]. Restoring the microbiota in these GF animals, especially through colonization with specific probiotic strains, can normalize these deficits. This highlights the critical role of gut microbes in providing necessary signals for optimal neurogenesis [116]. For example, administration of *Bifidobacteria* to GF mice significantly increased the numbers of proliferating neural stem cells and immature neurons in the dentate gyrus, suggesting that these bacteria are key drivers of AHN [112].

### 5.2. Bacterial Metabolites as Regulators of Neural Progenitor Cells

The influence of probiotics on neural progenitor cells is often mediated by bacterial metabolites, particularly SCFAs such as butyrate, propionate, and acetate. These molecules serve as crucial signaling agents in the gut–brain axis and can directly affect the proliferation, differentiation, and survival of neural progenitor cells (NPCs).

SCFAs, produced by the fermentation of dietary fibers by gut bacteria, can cross the BBB and exert direct effects on brain cells [118,119,120]. Butyrate, in particular, is a well-established histone deacetylase inhibitor [61,121]. By inhibiting histone deacetylases (HDACs), butyrate increases histone acetylation, which promotes a more open chromatin structure and enhances the transcription of genes involved in neuronal survival, synaptic plasticity, and memory formation [121]. This epigenetic mechanism enables butyrate to stimulate memory, synaptic plasticity, and, critically, neurogenesis [62].

Studies have shown that SCFAs can directly influence neural progenitor cell growth and differentiation. For example, in vitro studies exposing human neural progenitor cells to physiologically relevant concentrations of SCFAs demonstrated increased growth rates and elevated expression of proliferation-related genes, influencing neurodevelopmental gene expression [114,122]. This indicates a direct stimulatory effect of these bacterial metabolites on the cell cycle of NPCs. Furthermore, SCFA supplementation has been shown to prevent the reduction in neural stem cells and mature neurons induced by high-sugar diets, highlighting their protective and restorative role in neurogenesis [116]. The dynamic interaction between gut microbiota and SCFAs underscores their significant role in mediating the effects of environmental signals on brain function and neuroplasticity [123]. Gut microbiota-derived dietary metabolites can enter the systemic circulation and influence cell-to-cell interactions in the CNS, affecting cognitive function, mood, and behavior [124].

### 5.3. Effects of Probiotics on the Different Stages of Neurogenesis

Probiotics modulate adult neurogenesis by influencing multiple stages of the process; the proliferation of neural stem cells, their differentiation into mature neurons, the survival of these new neurons, and their functional integration into existing neural circuits. Probiotics consistently enhance the proliferation of neural stem cells and progenitor cells in the subgranular zone of the dentate gyrus. For example, *Bifidobacteria* colonization in GF mice significantly increased the numbers of proliferating neural stem cells and immature neurons [112]. This proliferation is often associated with the modulation of neurotrophic factors [51].

After proliferation, neural progenitor cells differentiate into various cell types, primarily neurons. Probiotic interventions, by impacting the microenvironment (such as reducing inflammation and increasing neurotrophic factors), promote the neuronal lineage differentiation of these progenitor cells [116]. This ensures that the newly generated cells develop into functional neurons rather than glial cells. The survival of newly born neurons is critical for their functional contribution. Probiotics, by reducing inflammation and oxidative stress and increasing neurotrophic support (such as BDNF), create a more favorable environment for the survival of immature neurons [51,52]. Studies have shown that probiotics can sustain hippocampal neural resilience and promote synaptic adaptability, which includes the survival of new neurons [51].

For new neurons to contribute to brain function, they must integrate functionally into existing neural networks by forming new synaptic connections. Although this stage is complex to assess, indirect evidence suggests a probiotic influence. The modulation of structural synaptic proteins and synaptic plasticity long-term potentiation/long-term depression (LTP/LTD) by probiotics strongly implies an impact on the successful integration of newly formed neurons [112]. By shaping host neural circuits, particularly during developmental windows, *Bifidobacteria* promote synapse formation, which is a prerequisite for neuronal integration [112]. Collectively, probiotics exert a comprehensive influence across the entire neurogenic cascade, from the initial birth of new cells to their functional maturation and integration.

It should be noted, however, that this mechanistic understanding is derived almost exclusively from preclinical rodent models—particularly GF animals and antibiotic-treated mice—which represent extreme microbiological conditions without direct human clinical counterparts. Direct assessment of neurogenesis in the living human brain remains ethically and technically unfeasible, and the currently available peripheral biomarkers (notably serum BDNF) provide only indirect, correlational evidence of neurogenic activity. Furthermore, while GF mouse models are invaluable for establishing causal microbiota-neurogenesis relationships, they exhibit global immunological, endocrinological, and physiological alterations that extend well beyond the gut microbiota, precluding direct inference about isolated probiotic effects in a neurologically intact human host. These considerations do not diminish the mechanistic value of the preclinical evidence reviewed here, but they underscore that the comprehensive neurogenic influence attributed to probiotics across the neurogenic cascade remains a working hypothesis grounded in animal and in vitro data, whose translation to human clinical outcomes warrants prospective evaluation through neuroimaging and biomarker strategies.

### 5.4. Translational Studies and Peripheral Biomarkers of Probiotic-Modulated Neurogenesis

Translational studies bridging preclinical findings to human applications, particularly regarding probiotic-modulated neurogenesis, are still in their early stages. Directly assessing neurogenesis in the living human brain is challenging due to ethical and technical limitations. Therefore, researchers often rely on indirect measures and peripheral biomarkers. BDNF is a well-established peripheral biomarker strongly correlated with central nervous system health and neurogenesis. Several clinical and preclinical studies have shown that probiotic interventions can increase serum or plasma levels of BDNF [42,43,44,45]. A meta-analysis indicated that probiotic treatment improved cognitive impairment in patients and animals, often alongside increased BDNF levels [43]. The only neurotrophin researched in relation to the intestinal microbiota is BDNF [114]. These peripheral BDNF changes are considered reflective of altered neurotrophic support in the brain, potentially indicating enhanced neurogenesis.

Given the strong link between inflammation and impaired neurogenesis, peripheral inflammatory markers (e.g., C-reactive protein, IL-6, TNF-α) are also used as indirect biomarkers. Probiotic interventions that reduce systemic inflammation may, therefore, be inferred to create a more permissive environment for neurogenesis [51,125].

Changes in gut microbiota composition (e.g., increased diversity, abundance of beneficial bacteria) and circulating levels of microbial metabolites (e.g., SCFAs) in humans following probiotic intervention can serve as indirect indicators of MGB axis interaction, which is known to influence neurogenic processes [59,60]. Although these changes do not directly measure neurogenesis, they suggest a modulated environment that may support neurogenic processes. For example, correlations between the gut microbiome and the CNS indicate that microbiome-targeted interventions are promising adjunctive treatments [59].

Despite promising preclinical data, a significant translational gap remains. Human clinical trials primarily focus on behavioral and mood outcomes rather than direct measures of neurogenesis. While improvements in mood and cognitive functions have been observed, attributing these directly to enhanced neurogenesis in humans requires further research [60,126]. There is considerable reluctance to translate the concept of integrating new neurons to humans [9]. Future translational studies should incorporate advanced neuroimaging techniques (e.g., functional MRI to assess hippocampal activity or MRS to measure neurochemical changes), along with robust peripheral biomarker analysis and detailed microbiota profiling, to establish a clearer link between probiotic intervention, microbiota changes, and human neurogenesis. This will require more diverse populations to verify generalizability and therapeutic potential [127,128]. The complex interplay between probiotics, bacterial metabolites, immune modulation, and epigenetic mechanisms highlights their potential as novel therapeutic avenues for promoting adult neurogenesis and improving outcomes in affective disorders. Further rigorous translational research is essential to fully realize this potential.

## 6. Probiotics and Modulation of Synaptic Plasticity

The complex communication network between the gut microbiota and the brain, known as the MGB axis, has become a crucial area of research for understanding neuroplasticity and cognitive function. Probiotics are increasingly recognized for their potential to modulate various aspects of neuronal function, including synaptic plasticity, which is fundamental for learning, memory, and adaptive brain function. Probiotic interventions appear to influence these processes through several mechanisms, such as the modulation of structural synaptic proteins, regulation of neurotrophic factors, effects on neuronal morphology, and direct modulation of LTP and LTD.

### 6.1. Impact of Probiotic Interventions on Structural Synaptic Proteins

**Figure 1:**
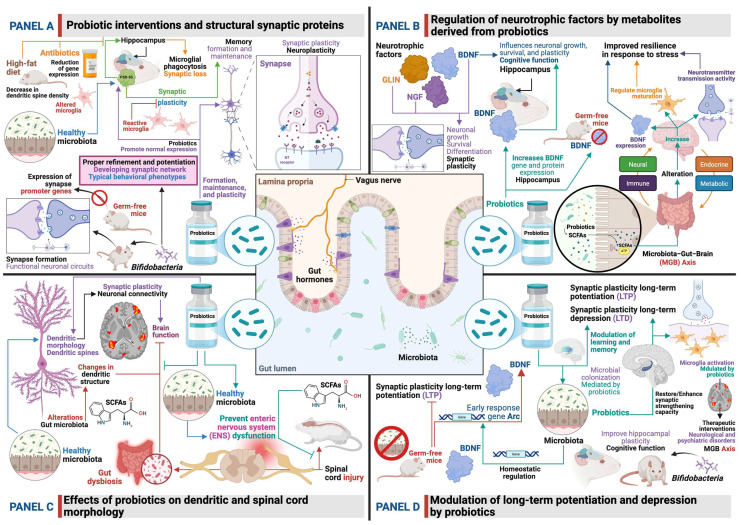
Probiotics and modulation of synaptic plasticity. (**Panel A**) Probiotic interventions and structural synaptic proteins: schematic showing how healthy microbiota and *Bifidobacteria* promote synapse formation and neuroplasticity, while antibiotics and a high-fat diet reduce dendritic spine density and impair synaptic maintenance through microglial phagocytosis. (**Panel B**) Regulation of neurotrophic factors by metabolites derived from probiotics: diagram illustrating how probiotics and SCFAs increase BDNF expression and synaptic protein levels in the hippocampus via neural, immune, endocrine, and metabolic pathways through the MGB axis. (**Panel C**) Effects of probiotics on dendritic and spinal cord morphology: illustration of how gut dysbiosis and altered microbiota composition affect dendritic structure and spine density, while healthy microbiota and SCFAs preserve ENS function and prevent spinal cord neuronal dysfunction. (**Panel D**) Modulation of long-term potentiation and depression by probiotics: diagram depicting how probiotics influence LTP and LTD through BDNF-mediated early response gene expression, microglial modulation, and homeostatic regulation, ultimately improving hippocampal cognitive function. Description in the text. Figure created with Created in Biorender. Muñoz-Carrillo, J.L. (2026) https://www.biorender.com (accessed on 5 February 2026).

Probiotic interventions have been shown to influence the expression and function of structural synaptic proteins, which are essential for synapse formation, maintenance, and plasticity. Synaptic plasticity involves physical and gene expression changes in neuronal synapses [112]. These changes are typically measured by alterations in the density and morphology of synaptic components. For example, some studies indicate that *Bifidobacteria* play a role in establishing functional neuronal circuits during postnatal development by promoting synapse formation. In GF mice, a decrease in the early expression of synapse-promoting genes was observed, while markers of reactive microglia increased. However, by postnatal day 20, both conventionalized mice (those exposed to the microbiota) and mice colonized with *Bifidobacteria* showed normal synaptic density and neuronal activity. This suggests that *Bifidobacteria* contribute to the proper refinement of the developing synaptic network and the potentiation of appropriate contacts, leading to circuit maturation and typical behavioral phenotypes (Figure 1A) [112].

Another study highlighted a reduction in the expression of genes related to synaptic plasticity, such as postsynaptic density protein 95 (PSD-95) and synaptophysin, in the hippocampus due to antibiotic administration [129]. These proteins are vital for memory formation and maintenance. This suggests that a healthy gut microbiota is crucial for maintaining the expression of these proteins and, by extension, for synaptic integrity. Probiotic interventions, by restoring microbial balance, could counteract these negative effects and promote the normal expression of these structural proteins. An important finding is that activated microglia, responsible for excessive phagocytosis of synaptic material, can contribute to impaired neuroplasticity [130]. A decrease in dendritic spine density was observed in the hippocampus of mice on a high-fat diet, possibly due to an increased rate of synaptic pruning, along with an increase in the PSD-95 deposition area in Iba1+ cells, indicating altered microglial activity toward synaptic components [130]. Probiotics, by modulating inflammation, could indirectly influence this microglial activity and thus protect synaptic structures (Figure 1A).

Furthermore, psychoactive bacteria, specifically *Bifidobacterium longum* Rosell^®^-175 and *Lactobacillus rhamnosus* JB-1, have been shown to affect the expression of brain proteins involved in metabolic and immunological processes in mice [131]. While the direct impact on specific structural synaptic proteins such as PSD-95 or synaptophysin was not detailed in the extract, the broader modulation of brain proteomes suggests an influence on synaptic architecture and function. The ability of the gut microbiota, and by extension probiotics, to influence synaptic activity in hippocampal neuronal circuits has been confirmed in systematic reviews, which point to various mechanisms, including the modulation of neurotrophins and neurotransmitter receptors (Figure 1A) [132].

### 6.2. Regulation of Neurotrophic Factors by Metabolites Derived from Probiotics

Neurotrophic factors, such as BDNF, nerve growth factor (NGF), and glial cell lineage-derived neurotrophic factor (GLIN), are crucial for neuronal growth, survival, differentiation, and synaptic plasticity. Probiotics and their metabolites play an important role in modulating the biosynthesis and expression of these factors. BDNF, in particular, is a key neurotrophin that influences neuronal growth, survival, and plasticity—processes essential for cognitive function [43]. Several studies have shown that probiotic use significantly increases BDNF gene and protein expression in the hippocampus [43]. This increase in neurotrophic factor biosynthesis is a key mechanism by which probiotics help maintain hippocampal neuronal resilience and promote synaptic adaptability [51]. Probiotics can promote the synthesis of BDNF, which enhances neuronal survival and differentiation [133]. GF animals typically exhibit a significant decrease in BDNF expression in both the cortex and hippocampus, and probiotic treatment can upregulate BDNF levels (Figure 1B) [113].

Probiotic-derived metabolites, such as SCFAs (butyrate, acetate, and propionate), are key mediators in MGB axis communication [114,134]. These metabolites can directly alter brain neurological function through vagal, endocrine, humoral, and immunological pathways, and can cross the BBB [134]. SCFAs have been shown to increase neurotransmitter transmission activity, BDNF expression, and regulate microglia maturation, thereby enhancing resilience in response to stress (Figure 1B) [135]. In vitro studies have demonstrated that exposing human neural progenitor cells to physiologically relevant concentrations of SCFAs can directly affect neuronal growth [114]. Furthermore, several *Lactobacillus* species have been reported to exhibit significant neuromodulatory effects, influencing neurotransmitters (including monoamines and 5-HT) and BDNF [134]. The impact of probiotic supplementation on serum BDNF levels has been studied, and meta-analyses indicate its potential to modulate this critical neurotrophic factor [42,44,45]. These findings underscore the profound influence of probiotic-derived metabolites on the availability and activity of neurotrophic factors essential for brain health and plasticity.

### 6.3. Effects on Dendritic and Spinal Cord Morphology

Dendritic morphology, including dendritic branching and the density and shape of dendritic spines, is directly related to synaptic plasticity and neuronal connectivity. Changes in dendritic structure can significantly affect brain function. The gut microbiota and probiotic interventions have been shown to influence these morphological aspects (Figure 1C).

The gut microbiota can affect dendritic morphology and help maintain the density and branching of dendritic spines [136]. Dendritic spines are small protrusions on dendrites that form specialized compartments for synaptic connections and are highly plastic structures [137]. Mature dendritic spines have a mushroom-like appearance and form strong synaptic connections, while immature spines can serve as markers for future connections and develop into mature spines through activity-dependent potentiation [137]. Alterations in the gut microbiota can lead to changes in these morphological characteristics. For example, propionic acid, an SCFA, has been shown to induce the loss of dendritic spines through specific signaling pathways [138]. Conversely, beneficial microorganisms and their metabolites can enhance dendritic spine density and branching, suggesting a dynamic interaction (Figure 1C).

Research highlights the significant role of the gut microbiota in influencing neurological recovery after spinal cord injury [139,140]. Spinal cord injury often leads to gut dysbiosis, which can affect neurological recovery [139]. Bidirectional communication between the gut and the CNS, including the spinal cord, via the gut–brain/spinal cord axis, can promote the production of pro-inflammatory metabolites that create an unfavorable environment for cell survival and locomotor recovery [140]. Microbiome modulation, for example through dietary fiber intervention such as inulin, has been shown to prevent ENS atrophy and dysmotility in mice following spinal cord injury, and microbially derived SCFAs have been shown to prevent ENS dysfunction in injured mice [141]. While these studies primarily focus on the ENS and recovery after injury, they suggest that a healthy gut microbiota, potentially supported by probiotics, could indirectly promote spinal cord health and neuronal plasticity by mitigating inflammation and supporting neuronal survival (Figure 1C). The broader context of the MGB axis also includes the gut’s influence on neurogenesis, myelination, microglia morphology, and the structure and permeability of the BBB [101]. SCFAs, whose production is influenced by probiotics, play a fundamental role in modifying the BBB and in the function of microglia, neurons, and astrocytes [136].

### 6.4. Modulation of Long-Term Potentiation and Depression

LTP and LTD are forms of activity-dependent synaptic plasticity that represent the cellular mechanisms underlying learning and memory. Probiotics and the gut microbiota have been shown to modulate these crucial processes (Figure 1D). LTP is a process that strengthens synaptic connections and is mediated by the overexpression of immediate early response genes [112].

Studies in GF mice indicate that the co-overexpression of immediate early response genes and the LTP pathway can be affected by the absence of gut microbiota. For example, the expression of BDNF, which is crucial for LTP, requires the prior induction of the immediate early response gene Arc, and both are increased in GF cohorts, suggesting specific overexpression of the pathway in the absence of microbial colonization [112]. This implies that microbial colonization, potentially mediated by probiotics, plays a role in the homeostatic regulation of these pathways. In aging, LTP typically declines [113]. Treatment with gut *Bifidobacteria* has been shown to improve hippocampal plasticity and cognitive function in healthy adult rats, consistent with the modulation of LTP [113]. This suggests that probiotics may restore or enhance synaptic strengthening capacity. The influence of the gut microbiota on the synaptic activity of hippocampal neuronal circuits has been confirmed, encompassing various molecular mechanisms, such as the regulation of neurotrophins, neurotransmitters, and intracellular molecular processes essential for LTP and LTD (Figure 1D) [132].

The broader field of neuroplasticity, which includes LTP and LTD, is profoundly influenced by the MGB axis [51,115,142]. Probiotics help maintain hippocampal neuronal resilience and promote synaptic adaptability through mechanisms such as suppressing pro-inflammatory responses, increasing neurotrophic factor biosynthesis, and mitigating oxidative stress [51]. These mechanisms are crucial for the proper functioning and modulation of LTP and LTD. The gut microbiota can affect brain gene expression, neurotransmitters, and microglia, and dysbiosis can interfere with neurodevelopmental pathways and alter plasticity [142]. Microglia activation, which can be modulated by probiotics, may link gut dysbiosis to impaired plasticity [142]. Understanding how probiotics modulate synaptic plasticity offers promising avenues for therapeutic interventions in neurological and psychiatric disorders, underscoring the importance of the MGB axis for maintaining optimal brain function (Figure 1D).

An important translational caveat applies to the LTP and LTD findings discussed in this section: the electrophysiological measurements used to characterize synaptic plasticity in rodent models—including hippocampal slice recordings and in vivo field potential analyses—have no direct, noninvasive equivalent in human research. In clinical settings, synaptic plasticity can only be inferred indirectly through neuroimaging proxies such as fMRI-based connectivity analyses and DTI, modalities that reflect aggregate network-level properties rather than individual synaptic events. Neuroimaging biomarkers in depression patients reveal alterations in amygdala-prefrontal connectivity and hippocampal volume that are broadly consistent with the synaptic plasticity deficits characterized in rodent models; however, the mechanistic specificity with which probiotics modulate these circuit-level phenotypes in humans remains to be established. Additionally, the considerable interindividual variability in microbiota composition—itself a determinant of SCFA production and neurotransmitter precursor availability—implies that the magnitude and directionality of probiotic-induced LTP modulation may differ substantially across individuals, a dimension of heterogeneity that is absent in the genetically uniform, controlled-housing rodent preparations on which the current evidence base is largely founded.

## 7. Interaction Between Probiotics and Synaptic Receptor Expression

The reciprocal relationship between the microbiota and the nervous system has been established as a fundamental pathway for regulating brain function, including processes such as neurogenesis and synaptic plasticity [143]. Currently, evidence supports the involvement of probiotics—whether through their administration, abundance, or absence—in models of affective disorders, where, in most cases, they exert therapeutic effects by modulating neurochemical processes and the morphology of the central or peripheral nervous system.

Among the most frequently reported molecular mechanisms through which probiotics exert their effects is the modulation of the expression of genes involved in neurotransmission systems such as the glutamatergic, GABAergic, and, to a lesser extent, monoaminergic systems (DA, 5-HT and NE). These systems have been shown to be associated with the regulation of affective states. Additionally, modulation of intracellular signaling pathways has been observed, including those associated with BDNF, Janus Kinase/Signal Transducer and Activator of Transcription (JAK/STAT), or Akt [144].

In this regard, the following subsections describe the effects of probiotic interventions on these systems, particularly those related to the expression of various neurotransmitter receptors and the intracellular signaling pathways implicated in affective disorders.

### 7.1. Modulation of Glutamatergic Signaling Through N-Methyl-D-Aspartate (NMDA) Receptors

The glutamatergic neurotransmission system is one of the main regulators of neuronal excitability, synaptic plasticity, and processes related to memory and learning [145]. NMDA receptors, among the most abundant in this system, play an essential role in synaptic activity; therefore, alterations in their expression or functionality have been associated with various affective disorders, especially those involving cognitive impairment [146]. In recent years, some studies have suggested that probiotic interventions may influence these glutamatergic mechanisms by modulating the expression of their receptors; however, the available evidence remains limited.

Given the importance of the glutamatergic system, it has been observed that certain probiotic interventions can modify specific components of this system. A study in Swiss albino male rats showed that oral administration of *Lactobacillus fermentum* ATCC 9338 for 28 days reversed depressive-like behaviors induced by the chronic unpredictable mild stress (CUMS) model. This effect was associated with a decrease in the expression of the N–methyl–D–aspartate Receptor 2B (NMDAR2B) subunit. Additionally, the treatment increased the expression of the glucocorticoid receptor, suggesting interactions between glutamatergic regulation and stress-response mechanisms [147].

In this context, and considering that several affective disorders involve cognitive alterations associated with dysfunctions in glutamatergic neurotransmission, some studies have investigated whether probiotics may support the recovery of processes such as memory and learning through the regulation of NMDA receptor expression. An example of this is a study conducted in male C57BL/6 mice with lead-induced cognitive deficits, in which oral administration of *Lactobacillus rhamnosus* GR-1 for five weeks improved cognition. This improvement was associated with a significant increase in the expression of N–methyl–D–aspartate Receptor 1 (NMDAR1) and NMDAR2B in the hippocampus, suggesting a functional restoration of this region, which is essential for synaptic plasticity [148].

Complementary to this, interventions combining probiotics and prebiotics have also been evaluated with the aim of enhancing their effects on the glutamatergic system. For example, in a model of obesity-associated cognitive impairment in male Sprague–Dawley rats, administration of *Enterococcus faecium* together with agave inulin for four weeks resulted in improvements in spatial and working memory, as well as increased hippocampal neurogenesis. These effects were associated with an increase in the expression of the N–methyl–D–aspartate Receptor 2A (NMDA2A) and NMDA2B subunits in the hippocampus, indicating a positive modulation of the NMDA receptor even under adverse metabolic conditions [149].

Taken together, although the current evidence remains limited, it suggests that probiotics may modulate the expression of different NMDA receptor subunits, thereby contributing to the recovery of altered cognitive and affective processes. It is important to note that further research is needed to accurately determine the role of probiotics in the regulation of glutamatergic receptors and to clarify the specific cellular mechanisms involved, as existing studies are still scarce. Nonetheless, available findings point toward a potential influence of microbial interventions on glutamatergic neurotransmission.

### 7.2. Effects on GABAergic Neurotransmission

Available evidence suggests that probiotic interventions can significantly regulate the GABAergic system, specifically through the modulation of GABA_A_ and GABA_B_ receptor expression. Although the precise mechanisms are not yet fully elucidated, studies in several animal models indicate that these microorganisms can promote differential changes depending on the species, probiotic strain, and the physiopathological conditions evaluated. For example, in a study using male C57BL/6 mice, administration of the probiotic NVP1704—composed of *Lactobacillus reuteri* NK33 and *Bifidobacterium adolescentis* NK98 (orally for five days)—reduced anxiety- and depression-like behaviors and increased sleep duration. These effects were associated with an increase in the expression of the α1 and α2 subunits of the GABA_A_ receptor in the prefrontal cortex and thalamus [150]. This highlights the ability of certain probiotics to modulate key brain regions involved in affective regulation and sleep.

In another study, findings from a juvenile Western Albino rat model of autism showed that three weeks of oral administration of *Bifidobacterium infantis*, *Lactobacillus bulgaricus*, or the commercial mixture PROTEXIN^®^ resulted in a significant increase in GABAergic receptor expression, with *Bifidobacterium infantis* showing the strongest effect. This modulation was accompanied by reduced intestinal permeability and oxidative stress. Although the model focused on autism rather than affective disorders, evidence indicates that such disorders also present GABAergic alterations, oxidative stress, and gut–brain axis dysregulation [151], suggesting a shared mechanism underlying different neuropsychiatric conditions [152]. Similarly, in stressed C57BL/6J mice, 40 days of oral administration of *Lactobacillus plantarum* SNK12 increased messenger RNA (mRNA) expression of GABA_A_ and GABA_B_ receptors, along with increased bdnf expression in the hippocampus, contributing to the attenuation of molecular effects of stress [153]. These findings link GABAergic modulation to neurotrophic factors essential for synaptic plasticity.

The modulatory effect of probiotics on this neurotransmission system has also been demonstrated in other species. For example, in zebrafish—an animal model widely used in preclinical anxiety research—administration of *Lactobacillus plantarum* increased the expression of the gabra1 gene, corresponding to the α1 subunit of the GABA_A_ receptor. This was associated with reduced anxiety-like behaviors and protection against stress-induced dysbiosis [154], suggesting that the ability of probiotics to modulate GABAergic neurotransmission is evolutionarily conserved and may represent a broad microbial mechanism across species.

An important aspect highlighted in this review is that not all studies rely on direct probiotic administration. Some have examined effects arising from changes in the intestinal abundance of specific strains following other interventions. For example, in a study using Sprague–Dawley rats with acute brain injury, increased levels of *Lactobacillus helveticus* after six days of ginsenoside Rb1 administration led to upregulation of the α2, β2, and γ2 subunits of the GABA_A_ receptor, as well as increased GABA_B_ expression in the hippocampus and striatum. The functional relevance of this modulation was confirmed through bicuculline administration, which attenuated the observed neuroprotective effects. Likewise, isolated administration of *Lactobacillus helveticus* for 15 days reproduced the same effects, reinforcing its role as a direct modulator of the GABAergic system [155].

Additionally, the GABAergic properties of different bacterial strains have been explored through in vitro studies, showing that several possess the gadB/gadC genes, which encode the enzyme glutamate decarboxylase (GAD), responsible for synthesizing GABA. Notably, *Lactobacillus brevis* Lb85 showed high efficiency in GABA production, while *Lactobacillus acidophilus* was the only strain presenting the gadBC system, demonstrating relevant differences between strains [156]. Recently, administration of *Lactobacillus reuteri* for four weeks in a murine model of autism induced by heterozygous deletion of the Cntnap2 gene resulted in increased expression of the γ subunit of the GABA_A_ receptor in the ventral hippocampus, which had been reduced by the model. This finding highlights the probiotic’s ability to restore GABAergic alterations associated with complex neurobehavioral conditions [157].

Finally, it is important to note that not all studies report beneficial effects. In young rats with dysbiosis induced by eight days of ampicillin administration, severe microbiota disruption was associated with depressive-like behaviors and impaired spatial memory, along with increased expression of the γ and δ subunits of the GABA_A_ receptor in the hippocampus, which intensified tonic neuronal inhibition. This suggests that maintaining microbiota balance—rather than merely increasing specific probiotics—is essential for proper GABAergic regulation [158]. Taken together, these studies reveal that probiotics can differentially modulate the GABAergic system depending on strain, species, physiopathological model, and experimental conditions. Unlike the glutamatergic system, where evidence remains more limited, the growing number of studies focusing on GABAergic neurotransmission may be explained by its central role in emotional regulation, its direct involvement in affective disorders, and the demonstrated ability of several gut bacteria to synthesize GABA. This functional convergence likely explains why the GABAergic system has become the most extensively studied target at the intersection of probiotics and mental health.

### 7.3. Interaction with Monoaminergic Systems (Serotonin, Dopamine, Norepinephrine)

Evidence regarding the influence of probiotics on monoaminergic systems (such as serotonergic, dopaminergic, and noradrenergic pathways) remains limited, even though these systems play a fundamental role in the pathophysiology of all affective disorders [159]. Nevertheless, the available studies provide an initial overview of how microbial modulation—through probiotic intervention or manipulation—may influence the expression of key receptors within these systems, revealing both specific and differential patterns of action across strains and experimental models.

For example, in a recent study using Wistar rats with depression-like behaviors induced by chronic stress, administration of the probiotic Bactolac—a mixture of *Lactobacillus plantarum* NBIMCC 8767 and *Streptococcus thermophilus* NBIMCC 8258 (for 8 weeks)—reduced depressive behaviors, which was associated with decreased expression of several receptors, including 5-Hydroxytryptamine 1A receptor (5-HT_1A_), dopamine receptor D1 (DRD1), alpha-2A adrenergic receptor (ADRA-_2A_), cannabinoid receptor type 1 (CNR1), and mineralocorticoid receptor (NR3C2). This study is particularly relevant given that these receptors are involved in affect regulation and in biological mechanisms disrupted in depression [160]. The broad set of pathways modulated suggests that certain probiotics may act simultaneously on multiple monoaminergic systems, consistent with the complex microbiota–brain interactions underlying affective disorders.

Similarly, strain-dependent effects were confirmed in a study using C57BL/6J mice, where dietary supplementation with *Enterococcus faecalis* EC-12 (for 4 weeks) reduced anxiety-like behaviors and increased expression of β3-adrenergic receptor (Adrb3) and vasopressin 1a receptor (Avpr1a). However, this strain did not significantly affect dopaminergic (Drd5) or serotonergic (Htr2b) receptors. These results reinforce the idea that probiotic effects are strain-specific and that not all strains induce widespread changes in receptor gene expression; rather, they may target specific components within monoaminergic systems [161].

Likewise, in male C57BL/6J mice subjected to restraint stress, administration of *Bacillus clausii* and *Lactobacillus fermentum* NMCC-14 for 7 and 14 days increased serotonin and norepinephrine levels, in addition to upregulating D1 and D2 receptor expression in the hippocampus and prefrontal cortex. These findings align with the literature highlighting the relevance of dopamine and norepinephrine in motivation, attention, and stress responses—processes commonly disrupted in affective disorders [50].

Furthermore, as with research on other neurotransmission systems, in vitro methodologies have expanded understanding of probiotic effects on components of the serotonergic system—one of the most studied systems in relation to affective disorders such as depression [162]. For instance, in a study using human intestinal epithelial cell lines HT-29 and Caco-2, exposure to *Lactobacillus acidophilus* and *Bifidobacterium longum* significantly increased expression of the 5-HT transporter (SERT) at both mRNA and protein levels [49]. Since SERT is the main target of several antidepressants, including selective serotonin reuptake inhibitors (SSRIs), the ability of certain probiotics to modulate its expression indicates a potential clinically relevant mechanism, not only for irritable bowel syndrome (IBS) but also for affective disorders involving serotonergic dysfunction.

Overall, the reviewed literature suggests that monoaminergic modulation through probiotics is real but variable. The evidence appears to lean toward a greater influence on dopaminergic and adrenergic receptors (D1, D2, DRD1, Adrb3, ADRA-2A), whereas studies specifically examining serotonergic receptors remain scarce, even though this system is central in depression and anxiety. These differentiated effects may reflect several key factors: (1) research on probiotic effects on serotonin has focused primarily on extracellular levels or SERT, rather than on receptor expression; and (2) strains studied thus far may exert more pronounced effects on dopaminergic and adrenergic pathways. Further research is needed to determine whether these modulations are consistent across bacterial species, whether they depend on treatment duration or the physiological condition of the animal model, and how these changes translate clinically in humans. In this regard, future studies should not only expand investigation into multiple serotonergic receptors but also elucidate the intracellular mechanisms—primarily at the signaling level—through which probiotics promote these modifications in monoaminergic systems.

### 7.4. Intracellular Signaling Pathways Involved

Another mechanism explored, though to a lesser extent, regarding the effects of probiotics on affective disorders involves the modulation of intracellular signaling pathways related to neuroplasticity, inflammation, and neuronal survival. Even though these mechanisms are not yet fully elucidated, emerging findings highlight specific pathways through which microbial interventions influence brain functions relevant to affective disorders.

In a study conducted by [163], *Lacticaseibacillus rhamnosus* JB-1 and *Limosilactobacillus reuteri* 6475 (administered for 28 days) decreased the expression of key genes in the JAK/STAT pathway, specifically JAK2, indicating a modulatory effect on central immune responses. Both probiotics also produced changes in the expression of BDNF and serotonergic-related genes, suggesting a combined interaction between neurotrophic and immunological pathways.

More recently, reference [19], using an in vitro model with neuronal (PC12) and monocytic (THP-1) cells, demonstrated that supplementation with a probiotic mixture composed of *B. bifidum* novaBBF7, *B. longum* novaBLG2, and *L. paracasei* TJB8 markedly increased BDNF expression. This increase was associated with elevated levels of p-Akt, supporting the involvement of the BDNF/TrkB–PI3K/Akt pathway as a central mechanism in promoting neuronal survival.

Although information regarding probiotic actions on intracellular signaling pathways implicated in affective disorders is still very limited, the evidence presented in this review suggests that probiotics may engage critical intracellular routes such as PI3K/Akt, JAK/STAT, and BDNF/TrkB through mechanisms ranging from neuroprotection to immunomodulation—all of which can influence affective states.

Translating these intracellular signaling findings to clinical contexts requires careful epistemological caution. The studies reviewed in this section were conducted predominantly in male rodent models and in cell lines (PC12, THP-1) that, while useful for pathway characterization, do not recapitulate the complexity of the human BBB, the pharmacokinetics of bacterially derived metabolites crossing into the central nervous system, or the sex-dependent hormonal modulation of neurotrophic and inflammatory signaling that is highly relevant to affective disorders. Specifically regarding the BBB, although SCFAs such as butyrate and acetate have been shown to cross the rodent BBB, the kinetics of SCFA transport in humans are conditioned by dietary composition, hepatic first-pass metabolism, and individual variation in gut microbiota fermentative capacity—factors that are tightly controlled in rodent laboratory settings but represent a major source of variability in clinical populations. Furthermore, the absence of comprehensive biomarker collection in current clinical trials—including those targeting the PI3K/Akt and BDNF/TrkB pathways—makes it currently impossible to verify whether the intracellular signaling changes documented in preclinical systems are reproduced at clinically meaningful levels in human subjects receiving probiotic supplementation.

Beyond intended neuroplasticity effects, the data reveal important off-target and strain-dependent adverse or null effects that must be considered for therapeutic applicability. First, not all probiotic interventions yield neuroplasticity benefits: *Enterococcus faecalis* EC-12, despite reducing anxiety-like behavior, produced no significant changes in dopaminergic (Drd5) or serotonergic (Htr2b) receptors [161], illustrating that beneficial behavioral outcomes do not necessarily imply broad receptor-level neuroplasticity. Second, dysbiosis-inducing conditions can paradoxically upregulate specific receptor subunits in ways that intensify rather than attenuate pathological states: ampicillin-induced microbiota disruption was associated with increased expression of the gamma and delta subunits of the GABA_A_ receptor in the hippocampus, intensifying tonic neuronal inhibition and worsening depressive-like behavior and spatial memory [158], a finding that underscores that indiscriminate microbiota modulation without strain specificity can produce off-target neurochemical effects antithetical to the intended therapeutic goal. Third, propionic acid, an SCFA, has been shown to induce dendritic spine loss via MAPK/ERK signaling and autophagic dysregulation [138], highlighting that individual microbial metabolites, even within the broadly pro-neuroplastic SCFA class, can exert divergent structural effects depending on their concentration and cellular context. Collectively, these observations reinforce that grouping probiotic strains at the genus level obscures clinically meaningful differences in their receptor targets, metabolic outputs, and potential for off-target effects, and that future therapeutic applications must be guided by strain-specific mechanistic profiles of the kind synthesized in Table 2.

## 8. Underlying Molecular Mechanisms

The MGB axis, a bidirectional communication pathway between the intestinal microbiome and the CNS, plays a crucial role in modulating adult neurogenesis and synaptic plasticity. Probiotic interventions, which introduce health-promoting microorganisms, are increasingly recognized for their capacity to influence neural functions underlying learning, memory, and cognitive flexibility [51]. These effects are particularly relevant for understanding the pathophysiology and treatment of affective disorders, where alterations in neurogenesis and synaptic function are often observed. Probiotics exert their influence through a sophisticated array of molecular mechanisms, including epigenetic regulation, modulation of inflammatory pathways, signaling via SCFAs, and the production of neuroactive peptides by the microbiota.

### 8.1. Underlying Molecular Mechanisms Involved in the Effect of Probiotics on Neurogenesis

**Figure 2:**
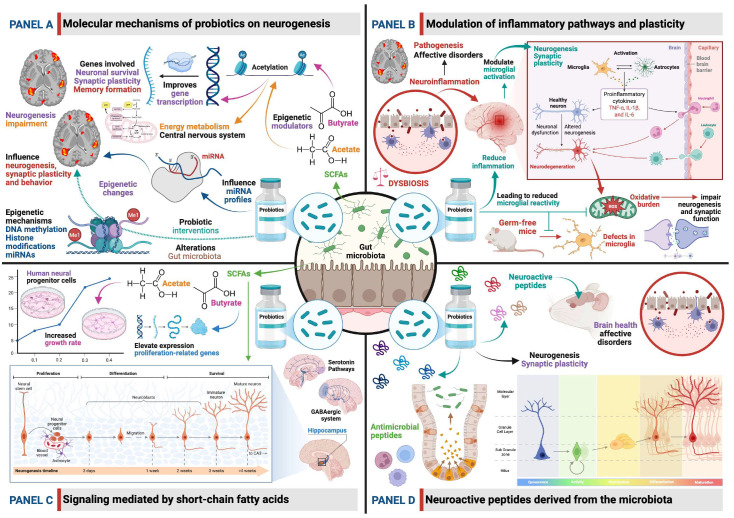
Underlying molecular mechanisms of probiotics. (**Panel A**) Molecular mechanisms of probiotics on neurogenesis: schematic showing how SCFAs, particularly butyrate and acetate, act as epigenetic modulators by inhibiting HDACs, increasing histone acetylation, and influencing DNA methylation and miRNA profiles, thereby promoting gene expression involved in neurogenesis, synaptic plasticity, and behavior. (**Panel B**) Modulation of inflammatory pathways and plasticity: diagram illustrating how dysbiosis activates pro-inflammatory pathways and microglial reactivity in GF conditions, impairing neurogenesis and synaptic function, while probiotics and prebiotics reduce microglial activation, decrease pro-inflammatory cytokines such as TNF-α, IL-6, and IL-18, and restore a neurogenic-supportive brain environment. (**Panel C**) Signaling mediated by short-chain fatty acids: illustration depicting how gut microbiota-derived SCFAs—acetate, propionate, and butyrate—cross the BBB and influence neuronal growth, neurotransmitter release, serotonin pathways, GABAergic signaling, and microglial homeostasis, promoting adult neurogenesis and synaptic adaptability. (**Panel D**) Neuroactive peptides derived from the microbiota: schematic showing how bacterially produced neuroactive and antimicrobial peptides interact with the nervous system, influencing neurogenesis, synaptic plasticity, and brain health in the context of affective disorders. Description in the text. Figure created with Created in Biorender. Muñoz-Carrillo, J.L. (2026) https://www.biorender.com (accessed 5 February 2026).

Epigenetic mechanisms, including DNA methylation, histone modifications, and microRNAs (miRNAs), are crucial regulators of gene expression without altering the underlying DNA sequence. These mechanisms are highly responsive to environmental cues, including those originating from the gut microbiota, and can profoundly influence neurogenesis and synaptic plasticity. The gut microbiota, and, by extension, probiotic interventions, can directly or indirectly interact with the host’s epigenome [63,64]. Microbial metabolites are increasingly recognized as significant inducers of epigenetic modifications (Figure 2A) [164]. For instance, alterations in gut microbiota composition can induce epigenetic changes that ultimately influence behavior [165].

SCFAs, particularly butyrate and acetate, are key microbial metabolites that act as epigenetic modulators [63,121,166]. Butyrate, a major SCFA produced by gut bacteria such as *Eubacterium*, *Clostridium*, and *Butyrivibrio*, is a potent histone deacetylase inhibitor [61,63]. By inhibiting HDACs, butyrate increases histone acetylation, promoting a more open chromatin structure and enhancing the transcription of genes involved in neuronal survival, synaptic plasticity, and memory formation [121]. This mechanism suggests that butyrate can stimulate memory and synaptic plasticity through epigenetic pathways [62]. Acetate can also cross the BBB and serve as a substrate for acetyl-CoA in neurons and glia, thereby influencing histone acetylation and energy metabolism in the central nervous system (Figure 2A) [121]. These SCFA-induced epigenetic changes can affect various physiological processes, including neurogenesis [61].

The gut microbiota also influences DNA methylation patterns, primarily through the production of methyl donors and modulators (e.g., folate producers) [121]. DNA methylation, typically occurring at CpG sites, can suppress gene transcription. Changes in methylation levels of genes involved in inflammatory responses have been linked to specific gut microbiota profiles (Figure 2A) [61]. Alterations in microbiota composition can lead to epigenetic changes that affect gene expression in the CNS, particularly in regions governing mood and neurological disorders [165]. While less directly explored in this context, miRNAs are small non-coding RNAs that regulate gene expression post-transcriptionally. There is an established link between the levels of specific miRNAs and the microbiota [61]. This suggests that probiotic interventions, by shaping the gut microbiota, could indirectly influence miRNA profiles in the brain, thereby affecting neurogenesis and synaptic plasticity (Figure 2A).

Overall, probiotics, by modulating the production of SCFAs and other metabolites, can trigger epigenetic changes that influence gene expression programs critical for adult neurogenesis and synaptic function. This epigenetic modulation offers a powerful mechanism through which the gut microbiome can shape brain development and function, impacting affective disorders.

### 8.2. Modulation of Inflammatory Pathways and Their Impact on Plasticity

Neuroinflammation, characterized by the activation of glial cells (microglia and astrocytes) and the release of pro-inflammatory cytokines, is a significant impediment to neurogenesis and synaptic plasticity, contributing to the pathogenesis of affective disorders [9,167]. Probiotics can exert anxiolytic and antidepressant effects by suppressing pro-inflammatory mechanisms and enhancing anti-inflammatory responses.

Microglia are the resident immune cells of the brain, and their activation state profoundly influences neurogenesis and synaptic function [168,169]. Gut microbiota provide essential cues to microglia, and dysbiosis can compromise intestinal barrier integrity, activate systemic immunity, and prime microglia toward a pro-inflammatory state [170]. Probiotics, such as *Lactobacillus rhamnosus* and *Bifidobacterium longum*, modulate microglial activation [51]. GF mice display defects in microglia, with altered cell proportions and an immature phenotype, indicating the gut microbiome’s role in microglial maturation [117]. Probiotic treatment can reverse antibiotic-induced decreases in neurogenesis and changes in monocyte populations, suggesting a role for monocytes and subsequent microglial activation in mediating gut microbiota–neurogenesis interactions [116]. Probiotics can rebalance microbial communities, enhance SCFA production, and reinforce intestinal barrier integrity, leading to reduced microglial reactivity (Figure 2B) [170].

Pro-inflammatory cytokines, such as IL-6 and TNF-α, inhibit adult neurogenesis in the hippocampus, linking inflammation to depression [53]. Conversely, anti-inflammatory cytokines promote neurogenesis. Probiotic administration reduces inflammation by attenuating the expression of inflammatory cytokines and microglial-induced inflammation, while simultaneously promoting the production of anti-inflammatory factors [52]. This shift in cytokine balance can alleviate neuroinflammation, thereby creating a more permissive environment for neurogenesis and synaptic plasticity (Figure 2B) [51,90]. For example, the probiotic VSL#3^®^ can modulate brain gene expression, including genes impacting inflammatory processes [171]. Chronic gastrointestinal inflammation, often mitigated by probiotics, is linked to altered hippocampal neurogenesis [172].

Beyond direct inflammation, probiotics also alleviate oxidative burden, which is often intertwined with inflammation and can impair neurogenesis and synaptic function [51]. Probiotic bacteria increase antioxidative activities while suppressing oxidative ones [52]. By modulating inflammatory pathways and microglial activity, probiotics help sustain hippocampal neural resilience and promote synaptic adaptability, offering a critical mechanism for their beneficial effects in affective disorders (Figure 2B).

### 8.3. Signaling Mediated by Short-Chain Fatty Acids

SCFAs such as acetate, propionate, and butyrate are crucial bacterial metabolites produced through the fermentation of dietary fiber in the gut [114,118]. These molecules are key mediators in microbiota–gut–brain communication because they can cross the BBB and influence brain function [118,120]. SCFAs play a multifaceted role in modulating neurogenesis and synaptic plasticity.

SCFAs directly affect neuronal growth and development. In vitro studies have shown that physiologically relevant concentrations of SCFAs increase the growth rate and elevate the expression of proliferation-related genes in human neural progenitor cells, indicating a direct effect on neuronal growth (Figure 2C) [114,119]. They also promote the maturation of glial cells and astrocytes [62]. SCFAs can promote neurogenesis. For example, a high-sugar diet reduces SCFAs, damages the BBB, and decreases neural stem cells and mature neurons—effects that can be reversed by SCFA intervention [116]. Butyrate production, in particular, has been shown to stimulate an increase in the number of hippocampal neurons (Figure 2C).

Butyrate acts as an HDAC inhibitor, leading to increased histone acetylation and enhanced gene expression crucial for neuronal survival and plasticity [61,121]. Acetate can also influence histone acetylation in the CNS [121]. SCFAs can influence the release of neurotransmitters. Butyrate affects the release of serotonin from intestinal enterochromaffin cells [62]. Acetate can be incorporated into the GABA metabolic cycle in the hypothalamus and activate the HPA axis (Figure 2C). Propionate has a protective effect on the BBB by mitigating oxidative stress [62]. SCFAs are crucial for regulating adult microglial homeostasis [173]. They can reduce the inflammatory response of microglia and influence their activation [120]. Given that microbiota provide essential cues to microglia, and SCFAs are a key output, they play a vital role in this aspect of neuroimmune regulation [169]. The dynamic interaction between gut microbiota and SCFAs highlights their significant role in mediating the effects of environmental signals on brain function and neuroplasticity [123].

Collectively, these mechanisms function as an integrated cascade: dysbiosis reduces butyrate production, diminishing HDAC inhibition and suppressing histone acetylation at neuroplasticity-related loci, including Bdnf. Simultaneously, reduced SCFA availability compromises BBB integrity, facilitating LPS translocation, microglial activation, and pro-inflammatory cytokine release, which further suppress hippocampal LTP and neurogenesis—a self-reinforcing cycle that probiotic intervention disrupts at multiple points simultaneously.

### 8.4. Neuroactive Peptides Derived from the Microbiota

Beyond small molecules like SCFAs, the gut microbiota also produces a range of peptides that can interact with the host nervous system, modulating brain function and potentially influencing neurogenesis and synaptic plasticity (Figure 2D). This area, often referred to as neuromicrobiology, is an emerging facet of the gut microbiome’s influence [59]. Some bacteria produce peptides involved in quorum sensing (cell-to-cell communication) that can influence the growth of beneficial bacteria and maintain microbial community structure [59]. Interestingly, some quorum sensing peptides have been shown to selectively penetrate the blood–brain barrier and enter the mouse brain, suggesting a direct influence on the CNS (Figure 2D). Gut microbiota can modulate the expression of host gut peptides. For example, administration of *Lactobacillus plantarum* SBT2227 has been shown to promote sleep in Drosophila melanogaster through the induction of neuropeptide F, a homolog of mammalian neuropeptide Y (NPY) [59]. Neuropeptides are small proteinaceous substances produced by nervous and endocrine cells in the gastrointestinal tract that act on neural and non-neuronal cells [174]. The manipulation of gut peptides via psychobiotics represents a promising opportunity to target mental disorders [175].

The gut microbiota produces common neurotransmitters and neuromodulators of the nervous system [176]. While the direct production of complex neuroactive peptides by probiotics and their direct impact on neurogenesis is an active area of research, it is clear that the broader gut microbiome generates neuroactive metabolites that influence the nervous system [177]. Some neuropeptides, such as NPY, possess antimicrobial activity, and innate immunity-related peptides are expressed in the mammalian brain [178]. Although primarily recognized for immune functions, these peptides might also regulate nervous system functions. The observation that antimicrobial peptides have structural features similar to amyloids, and that proteins like β-amyloid also have antimicrobial potential, suggests a complex interplay between microbial presence, host immune response, and neurological outcomes (Figure 2D) [179].

The existence of potential bioactive neuropeptides produced by the human gut microbiota underscores a significant, yet still largely unexplored, mechanism through which probiotics could affect neurogenesis and synaptic plasticity, particularly in the context of brain health and affective disorders (Figure 2D) [174]. Further research is needed to fully elucidate the specific neuroactive peptides produced by different probiotic strains and their precise roles in modulating brain function.

Taken together, the molecular mechanisms of epigenetic regulation via SCFAs, modulation of neuroinflammatory pathways, SCFA-mediated signaling, and neuroactive peptide production constitute a biologically coherent and mechanistically plausible framework for probiotic-induced neuroplasticity. Nevertheless, it is essential to situate this framework within its evidentiary boundaries. The vast majority of the mechanistic evidence reviewed in this section comes from in vitro systems and preclinical rodent models under controlled laboratory conditions, including standardized diets, uniform genetic backgrounds, specific-pathogen-free or GF housing, and predominantly male animal cohorts. These conditions differ substantially from the microbiological, genetic, dietary, and hormonal heterogeneity characteristic of human populations with affective disorders. The BBB represents a particularly critical translational bottleneck: while butyrate, acetate, and propionate have been shown to cross the rodent BBB and exert epigenetic and neuroimmune effects in brain parenchyma, the extent to which orally administered probiotics generate sufficient systemic SCFA concentrations to produce similar central effects in humans—whose hepatic metabolism, colonocyte uptake, and intestinal transit time differ substantially from rodents—remains an open empirical question. The mechanisms reviewed in Section 8 should therefore be interpreted as candidate pathways whose human relevance is supported by biological plausibility and indirect clinical evidence (notably BDNF modulation and inflammatory biomarker changes), but whose direct contribution to the neuroplastic and clinical outcomes observed in probiotic trials has yet to be fully established through mechanism-specific human studies.

## 9. Emerging Clinical Evidence on the Effect of Probiotic Treatment on Mood Disorders and Brain Plasticity

The growing understanding of the MGB axis has brought probiotics into the spotlight as potential therapeutic agents for mood disorders and modulators of brain plasticity. While preclinical studies have provided compelling evidence, translating these findings into robust clinical outcomes remains an active and evolving area of research.

### 9.1. Clinical Trials with Probiotic Interventions in Mood Disorders

Clinical trials investigating probiotic interventions in mood disorders, particularly depression and anxiety, are increasingly common. These studies aim to assess the efficacy of specific probiotic strains or multi-strain formulations in reducing symptoms and improving mental well-being in human populations. Early studies in healthy volunteers showed promising results. For example, individuals who received daily probiotic supplementation for four weeks reported decreased cognitive reactivity to sad mood, a recognized risk factor for depression [125]. Similarly, a fermented milk product containing various bacterial strains (including *Bifidobacterium animalis*, *Streptococcus thermophilus*, *Lactobacillus bulgaricus*, and *Lactobacillus lactis*) administered for four weeks to healthy women altered activity in brain regions involved in the central processing of emotion and sensation [40]. Another study found that *Bifidobacterium longum* strain 1714 attenuated stress responses and enhanced cognition in healthy subjects while altering electroencephalographic activity [65].

More recently, research has extended to individuals with diagnosed mood disorders. Meta-analyses of randomized controlled trials suggest that probiotics can benefit patients with depression, with some trials identifying a positive impact on individuals with mild or moderate depression [126,180]. For example, a pioneering randomized controlled trial investigated the effects of short-term, high-dose probiotic add-on therapy in depressed patients, assessing depressive symptoms alongside gut microbial and neural changes [181]. This study highlighted a promising new treatment approach targeting the MGB axis [181]. Another randomized controlled trial examined the impact of a multi-strain probiotic on self-reported indicators of depression, anxiety, mood, and associated biomarkers [182].

However, it is important to note that while some studies support the efficacy of probiotic supplementation, meta-analyses have generally found only small pooled effects, and the positive effects in major depression are still considered preliminary [183]. The US clinical trials registry (clinicaltrials.gov) shows ongoing clinical studies involving prebiotic, probiotic, or oral fecal microbiota transplantation supplementation for various neurological and psychiatric conditions, indicating growing interest in this field [125].

More recent and methodologically robust meta-analyses have begun to refine the magnitude of these effects. A 2025 systematic review and meta-analysis by [184], encompassing 19 RCTs and 1405 participants with diagnosed depression, reported a significant reduction in both depressive (SMD: −1.76; 95% CI: −2.42, −1.10) and anxiety scores (SMD: −1.60; 95% CI: −2.83, −0.36) following supplementation with probiotics, prebiotics, or synbiotics [184]. Notably, reference [185] conducted a complementary meta-analysis of 23 RCTs restricted to clinically diagnosed psychiatric samples (*n* = 1401), finding that probiotics produced substantially larger effects in clinical populations (d = −0.73 for psychiatric samples) compared to community samples, while prebiotics did not reach statistical significance for either depression or anxiety [185]. These findings collectively suggest that the therapeutic signal of probiotic interventions may be stronger in clinical populations than earlier general-sample meta-analyses indicated, and underscore the need to distinguish between healthy volunteers and patients with confirmed depressive diagnoses when interpreting pooled effect sizes. Nonetheless, high heterogeneity across studies (I^2^ > 90% in both meta-analyses) continues to limit the generalizability of these conclusions, emphasizing the imperative for standardized protocols.

A particularly innovative approach to clinical translation involves precision psychiatry-informed sample enrichment strategies. Reference [54] conducted an 8-week double-blind, placebo-controlled RCT in which patients with MDD were specifically selected based on concomitant low-grade systemic inflammation (defined by elevated BMI ≥ 25 kg/m^2^ and high-sensitivity C-reactive protein ≥ 1 mg/L), with the aim of identifying a biological subgroup hypothetically more likely to respond to the anti-inflammatory properties of probiotic intervention [54]. Participants received adjunctive *Limosilactobacillus reuteri* or placebo added to stabilized antidepressant treatment. While no overall antidepressant effect was observed for *L. reuteri* as a single-strain intervention in this inflammatory subgroup, the study yielded a significant methodological contribution: formic acid—an SCFA with anti-inflammatory properties—emerged as a preliminary biomarker and potential mediator of treatment response, with blood formic acid levels correlating with symptom improvement in the probiotic arm [54]. This RCT represents an important proof of concept for SCFAs as mechanistic biomarkers in probiotic clinical trials, directly linking gut microbial metabolite profiles to neuropsychiatric outcomes and exemplifying how enrichment strategies may enhance signal detection in future adequately powered trials.

### 9.2. Correlation Between Changes in Gut Microbiota and Biomarkers of Brain Plasticity

While clinical studies on mood disorders often focus on symptom reduction, research is also emerging on the correlation between changes in gut microbiota composition and biomarkers of brain plasticity in humans. Neuroimaging techniques, such as fMRI and DTI, are being used to explore these correlations. For instance, a study in healthy women showed that a fermented milk product affected activity in brain regions involved in emotional attention and sensory processing [41]. Although this study did not find significant changes in fecal microbiota composition, it did demonstrate altered brain activity patterns. Other research has explored correlations between fecal microbiota diversity and neuroimaging signals in brain regions such as the hypothalamus, hippocampus, and caudate nucleus [41]. It is important to distinguish between causal mechanistic evidence—currently established primarily in preclinical GF and in vitro systems—and biomarker-based associational evidence available from human clinical trials. Peripheral measures such as serum BDNF, inflammatory indices, and SCFA profiles serve as practical proxies for neuroplastic activity but do not provide direct measures of neurogenesis or synaptic plasticity in the living human brain. Therefore, the interpretation of clinical trial findings should be framed within this evidentiary hierarchy.

Biomarkers such as BDNF are also being investigated. BDNF is a crucial neurotrophin associated with neuronal growth, survival, and synaptic plasticity. Some studies indicate that probiotic supplementation can modulate neurotrophic factors such as BDNF [186]. For example, secondary analyses of randomized controlled trials have specifically examined the effect of probiotic supplementation on cognition, related brain functions, and BDNF levels in patients with depression [46].

The most methodologically comprehensive evaluation of this relationship to date is a 2025 GRADE-based dose–response meta-analysis by [42], which systematically examined the effect of probiotic supplementation on serum BDNF levels across multiple clinical and preclinical studies [42]. Using fractional polynomial models to assess non-linear dose–response relationships, this analysis confirmed a statistically significant probiotic-induced increase in circulating BDNF, while also identifying critical moderating variables, including probiotic strain composition, intervention duration, and baseline BDNF status. Importantly, the dose–response analysis revealed that effects on BDNF were not monotonically linear, suggesting the existence of threshold and ceiling effects that may explain some of the inconsistency observed in earlier studies. These findings reinforce the role of BDNF as a tractable peripheral biomarker of probiotic-induced neuroplasticity, while simultaneously highlighting the methodological complexity involved in establishing optimal dosing parameters for psychobiotic interventions targeting brain plasticity pathways.

A comprehensive review emphasizes that the gut microbiome influences neuroplasticity through mechanisms such as microbial metabolites, immune modulation, neurotransmitter regulation, and hormonal signaling [115]. Changes in these mediators, affected by the gut microbiota, could serve as indirect biomarkers reflecting altered brain plasticity. However, direct and consistent human evidence linking specific gut microbial changes to measurable, non-invasive biomarkers of neurogenesis or synaptic plasticity such as specific molecular markers in cerebrospinal fluid (CSF) or blood remains challenging to fully establish.

### 9.3. Methodological Limitations of Current Studies

Despite promising findings, current clinical research on probiotics and mood disorders faces several methodological limitations that hinder the definitive establishment of efficacy and generalizability. Many published randomized controlled trials (RCTs) have relatively small sample sizes and significant methodological heterogeneity [187,188]. This makes it difficult to draw strong, generalizable conclusions about the effect of adjuvant probiotic treatment [42]. The low number of RCTs involving participants with major depressive disorder further limits the ability to generalize findings from meta-analyses [182,189].

There is a considerable lack of standardization across studies regarding probiotic strains, dosages, and administration protocols [20,188]. Different strains, even within the same species, can have entirely different effects, making direct comparisons and meta-analyses challenging [20,21]. Treatment duration also varies widely, typically ranging from 4 to 24 weeks [126]. Studies often use various neuropsychiatric assessments and self-reported parameters of symptomatology without consistent use of clinical diagnoses or screening for comorbidities [20,188]. The lack of standardized outcome measures for gastrointestinal and psychological symptoms hinders meta-analyses and cross-study comparisons [20].

Potential confounding variables such as patients’ age, genotype, diet, concomitant medications, and comorbidities can obscure the true effects of probiotic interventions [127,188]. Many studies combine probiotics with first-line antidepressant or antipsychotic treatments, which, while useful for evaluating adjunctive therapy, complicates isolating the specific effects of the probiotics [127]. Many studies lack comprehensive biomarker collection, making it difficult to elucidate the mechanisms of action or to evaluate the colonization of the gut by administered probiotics. Robust and consistent biomarker collection, including those for the immune system, HPA axis, serotonin system, and gut microbiome, is necessary for advancing understanding [127].

Common limitations include reporting bias, insufficient power due to small sample sizes leading to increased risk of false-positive findings, and inconsistent reporting of probiotic activity, administration vehicle, and treatment adherence [20,190]. Few studies include robust follow-up periods to ascertain the longevity of effects [126]. These limitations underscore the urgent need for larger-scale, double-blind, placebo-controlled RCTs with standardized protocols, comprehensive biomarker collection, and longer follow-up periods to draw more definitive conclusions [127,183].

Recent preclinical studies have substantially refined the mechanistic understanding of probiotic-induced neurogenesis while highlighting important model-specific constraints relevant to clinical translation. Using GF mice colonized with a defined three-strain probiotic consortium (*Bacillus subtilis* TO-A, *Enterococcus faecium* T-110, and *Clostridium butyricum* TO-A; ProB3), researchers demonstrated that probiotic colonization not only restored hippocampal neural stem cell proliferation to levels comparable to specific-pathogen-free (SPF) controls, but also resolved a previously uncharacterized maturation arrest phenotype in DCX-positive neuroblasts that had remained “stuck” at an intermediate differentiation stage in GF animals [191]. This finding adds mechanistic detail to the existing GF neurogenesis literature by distinguishing between deficits in proliferation and deficits in maturation—two neurogenic substeps that may be differentially susceptible to microbial influence and that are rarely dissociated in earlier preclinical work. Employing a maternal separation model, a well-validated early-life stress paradigm, researchers showed that a multi-strain oral probiotic (OB) normalized both neural stem cell (nestin-GFP+) and neuroblast (DCX+) populations in the dentate gyrus. These effects were accompanied by a shift in microglial phenotype from a pro-inflammatory priming state toward a resting morphology [192]. The molecular hypothesis advanced by these authors—that butyrate-mediated histone acetylation at the Bdnf gene locus may underpin the proneurogenic probiotic effect—provides a direct mechanistic bridge between the SCFA-epigenetic pathways and behavioral neurogenesis outcomes, representing a meaningful advance in mechanistic specificity relative to earlier descriptive preclinical reports.

Despite these advances, a critical and frank appraisal of the translational gap between rodent preclinical models and human clinical reality is essential for contextualizing the evidence presented in this review. Several structural and biological differences between rodent and human gut–brain systems impose fundamental constraints on direct extrapolation. The human gut–brain axis differs profoundly from the rodent axis in the degree of prefrontal cortical expansion and frontoinsular integration—regions central to human emotional regulation—that have no functional equivalent in common rodent model organisms [193]. This neuroanatomical divergence means that even when probiotic interventions robustly modulate hippocampal neurogenesis in mice, the translational relevance of this specific neurogenic endpoint to human depression pathophysiology remains uncertain, given the ongoing scientific debate about the functional significance of AHN in humans [9]. Furthermore, it has been estimated that approximately 90% of drugs for mental disorders that demonstrate efficacy in preclinical trials ultimately fail in human clinical studies [194], a sobering statistic that reflects the composite challenge of species differences, model validity, microbiota composition divergence, and the complex heterogeneity of human psychiatric diagnoses. Practically, this translational challenge is further compounded by the fact that the vast majority of preclinical studies reviewed here used exclusively male rodents, a systematic bias that precludes assessment of sex-dependent differences in probiotic response—a significant gap given that affective disorders disproportionately affect women and that the gut microbiota demonstrates documented sex-hormone interactions [193]. The controlled housing conditions, standardized diets, and uniform genetic backgrounds of laboratory rodents further limit the generalizability of findings to the heterogeneous environmental, dietary, and microbiome-compositional landscapes characteristic of human populations [20]. Acknowledging these constraints does not diminish the value of preclinical mechanistic data, which remain indispensable for hypothesis generation and pathway identification, but it does underscore that each preclinical finding discussed in this review should be viewed as a mechanistic candidate requiring prospective validation in appropriately designed human trials rather than as a directly translatable therapeutic claim.

Recognizing this translational gap, the field is beginning to develop more human-relevant experimental platforms. A 2025 review specifically called for the integration of human induced pluripotent stem cell (iPSC)-derived neural models and organoid systems into psychobiotic research pipelines, arguing that these technologies provide a means to directly test the neurogenic and synaptic effects of probiotic-derived metabolites on human neural tissue in vitro, without the phylogenetic and anatomical confounds inherent in rodent models [194]. Additionally, precision psychobiotic discovery frameworks advocate the systematic use of humanized gnotobiotic models—GF mice colonized with defined human microbiota—as a bridging strategy that preserves mechanistic accessibility while improving translational fidelity [193]. These emerging methodological approaches represent a promising trajectory for the field, offering the possibility of iterative, mechanism-guided translation from bench to bedside that more conventional rodent-to-human pipelines have historically been unable to achieve in psychiatric drug development.

### 9.4. Interindividual Variability in Response to Probiotics

A significant challenge in probiotic research is the observed interindividual variability in response. Not every patient responds to probiotic interventions in the same way, and a particular intervention may be effective in one group but ineffective in another [20,188]. This variability arises from a complex interplay of host factors and microbial characteristics. The existing gut microbial composition of an individual plays a critical role. The efficacy of probiotics can be highly dependent on the underlying luminal microbial environment [64]. Individuals with gut dysbiosis, where the microbial composition differs from that of healthy individuals, may respond differently than those with a healthy baseline microbiome [20].

Genetic predispositions of the host can influence the gut microbiome and the response to interventions [187]. Dietary habits, lifestyle behaviors, and comorbidities can all modulate both the gut microbiota and the host’s response to probiotics [195]. For example, psychobiotic effects on anxiety can be modulated by lifestyle behaviors [195]. The efficacy of probiotics can also depend on the specific health status of the host (e.g., healthy individuals vs. those with diagnosed mood disorders) and other physiological parameters [190]. The effects of probiotics are highly strain-specific. Different strains, even of the same species, may have completely different effects on their hosts [20,21]. This means that benefits observed with one probiotic strain cannot be generalized to others [21]. Variations in the probiotic agents used, the dose, and the dosing pattern can significantly impact efficacy [64].

The ability of administered probiotics to survive passage through the gastrointestinal tract, colonize the gut, and interact with the existing microbiota can vary among individuals and products. This high degree of interindividual variability necessitates a more personalized approach to probiotic interventions. Future research should focus on identifying biomarkers that predict response to specific probiotic strains and tailoring interventions based on an individual’s unique microbial profile and host characteristics. This will require more diverse populations to verify generalizability and therapeutic potential [20].

The emerging field of precision microbiome medicine offers a compelling framework for addressing interindividual variability in probiotic response. A landmark 2025 review systematically examined the evidence for gut microbiome biomarkers as predictors of treatment response in depression, proposing a translational roadmap toward microbiome-informed precision psychiatry [196]. The authors identified several candidate microbial taxa and metabolic signatures—including reduced *Lactobacillus* and *Bifidobacterium* abundance, along with alterations in tryptophan and SCFA metabolic pathways—that distinguish treatment-responsive from treatment-resistant depressive profiles. They propose that multi-omics integration (combining gut metagenomics, metabolomics, and host transcriptomics) may enable prospective stratification of patients into microbiome-defined biological subtypes, analogous to precision oncology approaches, thereby guiding the selection of the most appropriate psychobiotic strain or consortium for each individual [196]. This perspective directly addresses the core challenge of interindividual variability in probiotic trials and represents a significant conceptual advance in the clinical translation of MGB axis-targeted interventions for affective disorders.

### 9.5. Conflicting Evidence, Null Results, and Cautionary Findings

A balanced appraisal of the evidence reviewed in this article requires explicit acknowledgment of null findings, negative results, and conflicting observations that constrain the otherwise promising narrative surrounding probiotics and neuroplasticity. At the clinical level, it is important to emphasize that meta-analyses of randomized controlled trials have consistently found only small pooled effect sizes for probiotic interventions in depression, and that positive effects specifically in patients with major depressive disorder—as opposed to subclinical or healthy populations—remain preliminary and are not yet sufficient to support definitive therapeutic recommendations [126,180,183]. Readers seeking quantitative synthesis of these pooled effects are directed to the meta-analyses cited, as a formal meta-analysis falls outside the methodological scope of this narrative review. At the receptor and molecular level, strain-dependent variability reveals important null effects: *Enterococcus faecalis* EC-12, despite producing measurable reductions in anxiety-like behavior, failed to induce significant changes in dopaminergic (Drd5) or serotonergic (Htr2b) receptor expression [161], illustrating that behavioral improvement does not necessarily reflect broad neuroplasticity at the receptor level and that mechanistic inferences from behavioral outcomes alone are insufficient. More critically, the finding discussed in Section 7.2—that antibiotic-induced microbiota disruption paradoxically upregulated the inhibitory gamma and delta subunits of the GABA_A_ receptor in the hippocampus, thereby intensifying tonic neuronal inhibition and worsening both depressive-like behavior and spatial memory [158]—demonstrates that microbiota modulation without strain specificity can produce neurochemical effects that are antithetical to the intended therapeutic goal. Within the SCFA literature, propionic acid—a bacterially produced SCFA—has been shown to induce dendritic spine loss via MAPK/ERK signaling and autophagic dysregulation [138], a finding that directly challenges the assumption that all microbial metabolites within the broadly pro-neuroplastic SCFA class exert uniform structural benefits on synaptic architecture. Finally, at the translational level, the persistence of probiotic-induced neuroplastic effects remains unresolved; a pilot study in patients with treatment-resistant depression reported recurrence of depressive symptoms following discontinuation of a probiotic consortium [190], raising the critical question of whether any neuroplastic benefits are maintained in the absence of continuous supplementation or represent transient, microbiota-dependent states. Taken together, these conflicting and cautionary findings underscore that the mechanistic and clinical evidence for probiotic-induced neuroplasticity, while biologically plausible and directionally promising, is not yet consistent enough across studies, strains, or populations to justify unqualified therapeutic claims, and that future research must report null and negative results with the same rigor applied to positive findings.

## 10. Synergistic Strategies: Probiotics Combined with Other Plasticity Modulators

The growing understanding of the MGB axis positions probiotics as promising modulators of adult neurogenesis and synaptic plasticity, which is particularly relevant for affective disorders. However, the complex nature of mental health conditions often requires integrated therapeutic approaches. Research increasingly explores synergistic strategies in which probiotics are combined with other established plasticity-enhancing interventions, aiming for more comprehensive and sustained therapeutic benefits. These multimodal interventions leverage diverse pathways that collectively optimize brain health and reduce symptoms associated with affective disorders.

### 10.1. Physical Exercise and Probiotics

Physical exercise is a well-documented enhancer of neurogenesis and synaptic plasticity, particularly within the hippocampus, a region critical for mood regulation and cognitive function [197,198]. It stimulates the proliferation and survival of new neurons and increases levels of BDNF, a key molecule supporting neuronal growth and plasticity [197,199]. Furthermore, physical activity exerts beneficial effects on the gut microbiota, increasing its diversity and richness, which in turn can improve metabolic and immunological responses [200,201,202].

Probiotics, in contrast, are known to impact the MGB axis. They can produce neuroactive molecules that directly or indirectly influence brain signaling [203]. Probiotics have also been shown to reduce biomarkers of oxidative stress and inflammatory cytokines [203]. Given that both interventions independently modulate the gut microbiota and can positively influence the CNS, combined interventions of exercise and probiotics are expected to more comprehensively and effectively address CNS diseases through the MGB axis, potentially outperforming single interventions [204,205]. While the explicit synergistic enhancement of specific brain mechanisms such as neurotrophic factors, neurotransmitter levels, and neuroinflammation by the combination of exercise and probiotics is an active area of research, studies are exploring how these combined interventions impact brain activity, including levels of neurotransmitters like GABA and glutamate, and inflammatory cytokines [206].

### 10.2. Specific Nutrients That Enhance Neuroplasticity (Omega-3 and Polyphenols)

Omega-3 polyunsaturated fatty acids (n-3 PUFA), particularly eicosapentaenoic acid (EPA) and docosahexaenoic acid (DHA), are vital for brain health. They have anti-inflammatory properties, optimize serotonergic transmission, and stabilize neuronal membranes, contributing to cognitive function and mood regulation [207,208]. Probiotics have demonstrated antidepressant-like effects and can modulate the dopaminergic pathway via the MGB axis [209]. Studies indicate that probiotics and n-3 PUFA can synergistically improve depressive behaviors, decrease stress hormones and inflammatory markers, and increase brain and gut concentrations of SCFAs and DA [209]. This combined approach suggests an enhanced impact on neurotransmitter balance and inflammatory responses.

Dietary polyphenols, abundant in plant-based foods, have antioxidant and anti-inflammatory properties and can stimulate molecules crucial for synaptic plasticity [210]. These compounds also function as prebiotics, fostering the growth of beneficial gut bacteria and the production of SCFAs [167]. A therapeutic regimen incorporating probiotics and natural polyphenols may offer advantages over classical pharmacological treatments because probiotics facilitate the production of diverse bioactive metabolites from dietary polyphenols. These metabolites can simultaneously ameliorate multiple risk factors associated with depression and anxiety [167]. Furthermore, the bioavailability of bioactive polyphenolic metabolites is greatly enhanced when administered alongside probiotics as a synbiotic [211]. This integrated strategy, by targeting multiple pathways including neurotransmitter modulation, neurotrophic support, inflammation control, and mitochondrial protection, presents a promising avenue for enhancing cognitive resilience and reducing depressive symptoms [212].

### 10.3. Environmental Enrichment and Cognitive Stimulation

Environmental enrichment, defined as complex living conditions that provide enhanced sensory, cognitive, motor, and social stimulation, is a potent modulator of brain plasticity. Environmental enrichment promotes AHN, improves memory and learning, and enhances synaptic plasticity [213]. Crucially, environmental enrichment can also modify gut microbiota composition and increase concentrations of beneficial microbial metabolites such as SCFAs, thereby improving brain plasticity and cognition [213,214].

The combination of probiotics with environmental enrichment represents a compelling synergistic strategy. While single lifestyle factors or microbiota modulation interventions may yield modest benefits, research demonstrates that environmental enrichment combined with specific probiotic interventions (e.g., *Bifidobacterium breve*) can amplify neuroprotective benefits and alleviate cognitive impairment by regulating the gut microbiota and microbial metabolites [213,214]. This suggests that the interplay between an enriched environment and targeted probiotic supplementation creates a powerful milieu for enhancing neuroplasticity and cognitive function, which is particularly relevant in neurodevelopmental and psychiatric disorders. The efficacy of specific probiotic strains may even be influenced by environmental variables such as physical activity and dietary factors [215].

### 10.4. Potential for Personalized Multimodal Interventions

The future of treating affective disorders is shifting toward personalized, multimodal interventions that consider each individual’s unique biological and environmental profiles [216]. The complex interplay between the gut microbiome, neuroplasticity, and mental health provides a strong rationale for integrating probiotics with other plasticity modulators as a framework for tailored therapeutic strategies [217].

Precision medicine, using multi-omic approaches—including genomics, transcriptomics, metabolomics, and gut microbiomics—aims to identify individual patient characteristics and enable the selection of the most effective treatments [216]. The MGB axis is increasingly recognized as a central target for such personalized interventions [218]. By combining probiotics with physical exercise, specific neuroplasticity-enhancing nutrients (such as omega-3 fatty acids and polyphenols), and environmental enrichment, interventions can be designed to target multiple molecular pathways and biological systems simultaneously. This comprehensive approach addresses the multifaceted nature of affective disorders and holds the potential to optimize adult neurogenesis and synaptic plasticity more effectively than single-target interventions. Further research is needed to determine the optimal combinations, dosages, and timing of these interventions to maximize their synergistic effects in clinical settings and to develop practical guidelines for their use in managing depression and enhancing brain health [212].

## 11. Translational and Therapeutic Considerations

The increasing understanding of the MGB axis has opened new avenues for treating affective disorders, positioning probiotics as promising modulators of neurogenesis and synaptic plasticity. To translate this potential into effective clinical strategies, it is necessary to carefully consider the design of probiotic consortia, the timing of intervention, the duration of treatment, and the identification of specific patient populations.

### 11.1. Designing Targeted Probiotic Consortia to Promote Neuroplasticity

The efficacy of probiotic interventions in modulating neuroplasticity and alleviating symptoms of affective disorders depends largely on the selection and combination of specific microbial strains. Not all probiotics are psychobiotics [219]. A precision psychiatry approach based on personalized probiotics is emerging as an ideal strategy, although current knowledge is still developing [220].

Research indicates that specific strains and consortia can influence key markers of neuroplasticity. Treatment with various *Bifidobacteria* strains has been shown to counteract the decline in BDNF levels [113], a key neurotrophin involved in dendritic spine formation, neuronal growth, and synaptic plasticity [113]. Elevated BDNF levels have been observed in studies with *Bifidobacterium infantis* [221] and other probiotics under conditions of chronic stress, inflammation, and aging, likely through reduced microglia activation [221]. Furthermore, probiotic mixtures have been shown to enhance certain forms of synaptic plasticity, such as LTP, which is typically reduced in older animals [113]. This suggests that treating age-related gut microbiota alterations with probiotics may restore hippocampal plasticity [113].

Additionally, probiotics have been reported to exert their effects through multiple molecular mechanisms, including improved intestinal barrier function, regulation of gut-associated lymphoid tissue, increased anti-inflammatory cytokines, modulation of vagus nerve activity, and regulation of neurotransmitter receptor expression (e.g., GABA) and the HPA axis [180]. These mechanisms collectively contribute to neuroplasticity. The complexity of the MGB axis necessitates the design of multi-strain probiotic consortia that can act synergistically on multiple pathways. For example, specific combinations of *Lactobacillus* and *Bifidobacterium* strains have been commonly used in studies [219]. Future research should focus on identifying the most potent microbial strains and optimal combinations to promote their growth and maximize their impact on brain function [221].

### 11.2. Optimal Time Windows for Intervention

Identifying the optimal time windows for probiotic intervention is crucial for maximizing therapeutic outcomes. The brain undergoes significant maturation during adolescence, a period marked by increased neuronal plasticity, making it particularly vulnerable to environmental influences such as stress [120]. This suggests that interventions in childhood or adolescence could be especially impactful, although the current neuropsychiatric literature contains few reports of probiotic trials during these critical developmental stages [190].

While most current trials focus on adult populations, many conducted in middle-aged adults [184], some studies have shown beneficial effects of probiotic supplementation on depressive symptoms in adults [219,222]. For example, administration of *Bifidobacterium breve* A1 for four weeks significantly reduced anxiety and depression levels [223]. Randomized, placebo-controlled clinical trials have demonstrated that probiotic regimens as short as 30 days can decrease stress levels, while longer periods (e.g., 8 weeks) produce reductions in depression scores [224]. Evidence points to potential benefits at different stages of life, but more research is needed to determine the specific periods when the developing or aging MGB axis is most susceptible to probiotic modulation for neuroplasticity.

### 11.3. Considerations Regarding Treatment Duration and Persistence of Effects

The duration of probiotic treatment and the persistence of its beneficial effects are important translational considerations. In most reviewed clinical trials of probiotics for neuropsychiatric outcomes, treatment duration has generally been less than 60 days, with limited assessment of long-term outcomes [190]. Some studies have demonstrated positive impacts of probiotic interventions, particularly in individuals with mild to moderate depression, with treatment periods ranging from 4 to 24 weeks [126].

However, whether probiotic colonization of the gut is permanent or only temporary remains largely unresolved [221]. The need for continuous use to maintain a healthy gut microbiota is often suggested [221]. Some evidence indicates that effects may not persist after treatment is discontinued. For example, a pilot study in patients with treatment-resistant depression reported recurrence of symptoms after discontinuation of treatment with a consortium of *L. acidophilus*, *B. bifidum*, and *S. thermophilus* [190]. This suggests that continuous or periodic probiotic supplementation may be necessary for sustained modulation of neuroplasticity and maintenance of therapeutic benefits. More comprehensive research, including extended follow-up periods, is needed to determine the duration of these effects [126].

### 11.4. Specific Populations That May Benefit Particularly

Probiotic interventions are particularly promising for certain populations in whom conventional treatments are insufficient or dysregulation of the HPA axis is more pronounced. For example, a significant proportion of patients with major depressive disorder do not respond to initial antidepressant treatments, with up to one-third exhibiting resistance [222]. In these cases, probiotics are emerging as a promising alternative or complementary therapy [181,222,225]. For instance, complementary probiotic therapy in patients with depression has demonstrated improvement in depressive symptoms, maintained microbial diversity, and increased the abundance of *Lactobacillus* [181]. This is crucial, as traditional treatments for depression, which primarily target the brain, are often insufficient for treatment-resistant individuals [225].

Depression in older adults is often undertreated [226]. While some reviews suggest that clinical trials have not shown significant results in people over 65 years of age [224], other systematic reviews have included older participants in studies examining psychobiotics for psychiatric and cognitive disorders [219]. Age-related changes in the gut microbiota have been linked to decreased BDNF and hippocampal plasticity, which could be reversed with probiotic treatment [113]. Given the role of probiotics in influencing the gut microbiota and BDNF, older adults, who may experience age-related decline in neuroplasticity, could benefit from targeted interventions.

Several studies have identified a positive impact of probiotic intervention in individuals with mild to moderate symptoms of depression and anxiety [126,227]. Probiotics may also reduce the risk of depression in healthy individuals [224], suggesting a role in prevention or early intervention for those with subclinical symptoms or risk factors. In conclusion, while the therapeutic potential of probiotics to modulate neuroplasticity in affective disorders is significant, further targeted research is needed to optimize probiotic consortia, define ideal intervention timeframes, establish long-term treatment strategies, and identify the patient groups with the greatest response. This will pave the way for microbiome-based precision therapies that can address the urgent need for alternative and more effective treatment approaches in mental health.

Despite their generally favorable tolerability, probiotics are not risk-free for all clinical populations with affective disorders. Immunocompromised patients, those with structural cardiac defects, or individuals with severe intestinal barrier disruption face a significant risk of bacteremia or fungemia following probiotic administration [228,229,230]. In the context of affective disorders, concomitant use of immunosuppressants or broad-spectrum antibiotics may unpredictably alter probiotic colonization and produce off-target neurochemical effects. Strain-specific adverse interactions with psychotropic medications remain largely uninvestigated and represent a critical knowledge gap that requires prospective pharmacovigilance in future clinical trials.

### 11.5. Fecal Microbiota Transplantation as a Comparator and Complementary Strategy to Probiotic Interventions

Fecal microbiota transplantation (FMT) has emerged as a promising approach for modulating the gut microbiota in neuropsychiatric disorders, and its comparison with probiotic interventions offers important translational insights. FMT involves transferring a complete fecal microbiota from a healthy donor to a recipient, aiming to restore microbial diversity and eubiosis. Unlike probiotics, which introduce a limited number of well-characterized strains, FMT provides a broader and more complex microbial reconstitution, potentially engaging a wider array of MGB axis pathways simultaneously. Notably, FMT has been shown to transfer depressive-like phenotypes between animals—transplantation of fecal microbiota from chronically stressed or depressed donor mice into healthy recipients reproduces depression-like behaviors along with significant reductions in hippocampal levels of BDNF, 5-HT, and norepinephrine—providing compelling mechanistic evidence that gut microbiota dysbiosis directly modulates neuroplasticity-related pathways [227]. Conversely, FMT from healthy donors has been shown to rescue depressive phenotypes and restore hippocampal BDNF expression, 5-HT levels, and neurogenesis markers in preclinical models of CUMS [231], and to increase hippocampal BDNF, vascular endothelial growth factor-A (VEGF-A), and NGF expression, along with enhanced neurogenesis in the DG [232].

In the clinical setting, the first double-blind, randomized controlled pilot trial evaluating FMT in patients with moderate-to-severe MDD demonstrated that the procedure was feasible, well accepted, and safe, with zero attrition over 8 weeks of follow-up, marking a significant milestone for microbiome-based psychiatric intervention [233]. Building on this, a recent meta-analysis of 12 RCTs with 681 participants found that FMT led to a significant reduction in depressive symptoms, with effects most pronounced in the short to medium term and diminishing at 6 months of follow-up [234]. Additionally, recent multi-omics analyses have shown that FMT can enhance the antidepressant effects of SSRIs by modulating tryptophan metabolism, neuroinflammatory pathways, and circadian gene expression in the hippocampus and prefrontal cortex [235].

However, important distinctions exist between FMT and probiotic interventions that affect their respective translational potential. Probiotics offer greater reproducibility, safety, and regulatory tractability, as their composition is precisely defined and their administration does not carry the immunological and infectious risks associated with donor-dependent FMT preparations [236]. Furthermore, the strain-specific mechanisms of action of probiotics allow for more targeted therapeutic hypotheses and clearer mechanistic interpretation of clinical outcomes. In contrast, the compositional complexity of FMT, while potentially advantageous for broad microbiota restoration, complicates the identification of the specific microbial or metabolic mediators responsible for its effects on neurogenesis and synaptic plasticity.

Rather than viewing these approaches as mutually exclusive, an emerging perspective positions them as potentially complementary. FMT may be used to establish a favorable microbial baseline in individuals with severe dysbiosis, after which targeted probiotic supplementation could help maintain and consolidate neuroplasticity-promoting microbial communities—a sequential strategy with significant potential for personalized psychobiotic medicine. Future research should include head-to-head comparisons of FMT and probiotic interventions in well-powered RCTs, incorporating neuroplasticity biomarkers such as BDNF, hippocampal volume, SCFA profiles, and inflammatory indices as primary or secondary outcomes, to establish the relative efficacy, safety, and optimal sequencing of these complementary microbiota-targeted therapeutic approaches in affective disorders.

## 12. Conclusions

The intricate interplay between the gut microbiome and the brain, mediated by the MGB axis, has emerged as a pivotal factor in the pathophysiology and treatment of affective disorders. This review has highlighted the compelling role of probiotics as modulators of adult neurogenesis and synaptic plasticity, offering novel perspectives on mental health. Preclinical evidence unequivocally demonstrates that specific probiotic strains, particularly *Lactobacillus* and *Bifidobacterium* species, can significantly influence hippocampal neurogenesis. These beneficial microorganisms and their metabolites, such as short-chain fatty acids, regulate neural progenitor cell proliferation, differentiation, survival, and integration into existing neural circuits. SCFAs, especially butyrate, exert epigenetic control by inhibiting histone deacetylases, thereby promoting gene expression crucial for neuronal development and plasticity. Moreover, probiotics modulate inflammatory pathways by suppressing pro-inflammatory cytokines and influencing microglial activity, creating a neurogenic-supportive brain environment. The production of neuroactive peptides by the microbiota further underscores its capacity to directly signal within the gut–brain axis, influencing neural functions.

Translational research, though still in its early stages, is beginning to bridge the gap between preclinical findings and human applications. RCTs investigating probiotic interventions in mood disorders show promising trends, with some studies indicating positive effects on symptoms of depression and anxiety. For example, a multi-strain probiotic demonstrated a positive effect on cognitive reactivity to sad moods in healthy adults, and an add-on probiotic treatment improved depressive symptoms in patients. These effects are often correlated with shifts in gut microbiota composition and observable changes in peripheral biomarkers such as BDNF, a key indicator of neuroplasticity. Neuroimaging studies are also beginning to reveal altered brain activity patterns following probiotic administration, suggesting direct impacts on neural processing. Despite these encouraging developments, the field faces significant methodological limitations. Challenges include small and heterogeneous sample sizes, lack of standardization in probiotic strains and dosages, inconsistent outcome measures, and the presence of confounding variables in clinical trials. Many studies use a variety of neuropsychiatric assessments without consistent use of clinical diagnoses or screening for comorbidities. A major hurdle remains the considerable interindividual variability in response to probiotic interventions, influenced by host factors such as baseline microbiota composition, genetics, diet, and lifestyle, as well as probiotic characteristics like strain specificity, dosage, and formulation. This variability underscores the need for personalized approaches and robust biomarkers to predict therapeutic efficacy. In conclusion, probiotics hold significant potential as innovative therapeutic agents for affective disorders by targeting adult neurogenesis and synaptic plasticity. Future research must prioritize larger, well-designed, placebo-controlled clinical trials with standardized methodologies, comprehensive multi-omics analyses, and long-term follow-up to elucidate the precise mechanisms and optimal applications. Advancements in identifying specific probiotic strains or consortia tailored to individual microbial profiles will pave the way for precision psychobiotic interventions, ultimately contributing to more effective strategies for the prevention and treatment of mental health conditions.

Translating the mechanistic evidence reviewed here into clinical practice requires a stepwise roadmap. In the short term, research should prioritize adequately powered, double-blind RCTs in clinically diagnosed populations using standardized strain-specific consortia, comprehensive biomarker panels—including serum BDNF, inflammatory indices, and SCFA profiles—and neuroimaging outcomes such as hippocampal volume and amygdala-prefrontal connectivity. In parallel, iPSC-derived neural organoid platforms and humanized gnotobiotic models should be integrated into preclinical pipelines to bridge the translational gap. In the medium term, multi-omics stratification frameworks should guide patient selection based on microbiome-defined biological subtypes, enabling precision psychobiotic interventions. Ultimately, sequential strategies combining FMT for microbiota restoration with targeted probiotic maintenance represent a clinically viable pathway toward personalized microbiome-based therapeutics for affective disorders.

## Figures and Tables

**Table 1:** Biomarkers of neuroplasticity reported in studies of affective disorders and probiotic interventions.

Biomarker Category	Specific Biomarker	Measurement Approach	Findings in Affective Disorders	Relationship to Probiotic Intervention	References
Neuroimaging Structural	Hippocampal volume	Structural MRI	Consistently reduced in MDD patients; indicative of impaired neurogenesis or neuronal atrophy	Not directly measured in probiotic trials; proposed as future outcome measure	[31,32]
Neuroimaging Structural	Amygdala volume	Structural MRI	Increased in patients with depression and comorbid anxiety; comorbid anxiety reduces depression-related structural changes	Not yet evaluated in probiotic RCTs	[32]
Neuroimaging Functional	Amygdala-prefrontal connectivity	fMRI/DTI	Reduced functional coupling in corticolimbic circuitry in anxiety and depression	Altered brain activity patterns observed after fermented milk product consumption in healthy women	[31,40,41]
Neuroimaging Functional	Hypothalamic, hippocampal and caudate nucleus activity	fMRI	Correlated with fecal microbiota diversity in healthy subjects	Preliminary evidence of altered activity following probiotic administration	[41]
Neuroimaging Functional	Personalized brain circuit scores	fMRI + machine learning	Identify clinically distinct biotypes in depression and anxiety	Proposed as future precision medicine tool	[38]
Neurotrophic Peripheral	Brain-derived neurotrophic factor (BDNF)	Serum/plasma ELISA	Reduced in MDD and anxiety; correlates with hippocampal atrophy and neurogenesis impairment	Increased by probiotic supplementation in multiple clinical and preclinical studies; dose–response relationship confirmed	[42,43,44,45,46]
Neurotransmitter Peripheral	Serotonin (5-HT)	Plasma/CSF assay	Dysregulated in MDD and anxiety; ~90% of body 5-HT synthesized in gut	Modulated indirectly via microbial tryptophan metabolism and SERT expression; *Lactobacillus acidophilus* and *B. longum* increase SERT expression	[47,48,49]
Neurotransmitter Peripheral	Dopamine (DA)	Plasma/brain tissue	Reduced in motivation and reward circuits in depression	Increased by *Bacillus clausii* and *L. fermentum* NMCC-14 in hippocampus and PFC	[50]
Neurotransmitter Peripheral	Norepinephrine (NE)	Plasma/brain tissue	Dysregulated in stress response and affective disorders	Increased alongside dopamine following probiotic treatment in restraint stress models	[50]
Inflammatory Peripheral	Interleukin-6 (IL-6)	Serum ELISA	Pro-inflammatory; inhibits hippocampal neurogenesis; elevated in depression	Reduced by probiotic administration; shift toward anti-inflammatory cytokine profile	[51,52,53]
Inflammatory Peripheral	Tumor Necrosis Factor-alpha (TNF-α)	Serum ELISA	Elevated in MDD; impairs neuroplasticity and synaptic function	Attenuated by probiotics via cholinergic anti-inflammatory pathway and gut barrier reinforcement	[51,52]
Inflammatory Peripheral	C-reactive protein (CRP)	High-sensitivity serum assay	Elevated in inflammatory subgroup of MDD; used for patient stratification	Used as inclusion criterion in precision psychobiotic RCT; correlates with treatment response	[54]
Metabolic Peripheral	Short-chain fatty acids (SCFAs): acetate, propionate, butyrate	Fecal/plasma metabolomics	Reduced in gut dysbiosis; deficiency associated with vulnerability to inflammation and depression	Increased by probiotic supplementation; formic acid (SCFA) emerged as preliminary biomarker of treatment response	[54,55,56,57,58]
Microbiota Compositional	Gut microbiota diversity and abundance (e.g., *Lactobacillus*, *Bifidobacterium*)	16S rRNA sequencing/metagenomics	Reduced diversity and *Lactobacillus*/*Bifidobacterium* abundance in depression; associated with altered tryptophan and SCFA metabolism	Restored by probiotic supplementation; changes correlate with mood and plasticity biomarker improvements	[20,59,60]
Epigenetic Indirect	Histone acetylation/DNA methylation profiles	Epigenomic assays (preclinical)	Altered at neuroplasticity-related gene loci (e.g., Bdnf) in stress models	Modulated by butyrate-mediated HDAC inhibition following probiotic intervention	[61,62,63,64]
Electrophysiological	Electroencephalographic (EEG) activity	EEG	Altered patterns associated with stress and mood dysregulation	Modified by *B. longum* 1714 in healthy subjects	[65]

**Table 2:** Strain-specific effects of probiotic interventions on neurogenesis and synaptic plasticity reviewed in this article. Strains are organized by neurotransmitter system primarily modulated.

Probiotic Strain/Consortium	Animal/Cell Model (Duration)	Neurogenesis-Related Effects	Synaptic Plasticity Effects	Receptors/Pathways	Refs.
*Lacticaseibacillus rhamnosus* JB-1	Mice (C57BL/6); in vitro monocytic cells (THP-1)	↑ BDNF gene expression; modulation of serotonergic genes	↓ JAK2/STAT expression; interaction with BDNF/TrkB pathway	GABAergic; serotonergic	[131,163]
*Bifidobacterium longum* Rosell^®^-175	Mice (C57BL/6)	Modulation of brain proteome involved in metabolic and immunological processes	Broad modulation of synaptic architecture; indirect via brain protein expression	Not specified (proteome-level)	[131]
*Limosilactobacillus reuteri* NK33 + *Bifidobacterium adolescentis* NK98 (NVP1704)	Male C57BL/6 mice	Reduced anxiety/depression-like behavior; ↑ sleep duration	↑ GABA_A_ receptor α1, α2 subunits in PFC and thalamus	GABAergic (GABA_A_ α1, α2)	[150]
*Lactobacillus plantarum* SNK12	Stressed C57BL/6J mice (40 days)	↑ BDNF mRNA in hippocampus; attenuation of molecular stress effects	↑ GABA_A_ and GABA_B_ receptor mRNA expression in hippocampus	GABAergic (GABA_A_, GABA_B_)	[153]
*Lactobacillus fermentum* ATCC 9338	Male Swiss albino rats; CUMS model (28 days)	Reversal of depressive-like behavior; ↑ glucocorticoid receptor expression	↓ NMDAR2B subunit expression in hippocampus	Glutamatergic (NMDAR2B)	[147]
*Lactobacillus rhamnosus* GR-1	Male C57BL/6 mice; lead-induced cognitive deficit (5 weeks)	↑ cognitive function; functional restoration of hippocampus	↑ NMDAR1 and NMDAR2B expression in hippocampus	Glutamatergic (NMDAR1, NMDAR2B)	[148]
*Enterococcus faecium* + agave inulin (synbiotic)	Male Sprague-Dawley rats; obesity-associated cognitive impairment (4 weeks)	↑ hippocampal neurogenesis; improved spatial and working memory	↑ NMDA2A and NMDA2B subunits in hippocampus	Glutamatergic (NMDA2A, NMDA2B)	[149]
*Bifidobacterium infantis*	Juvenile Western Albino rats; autism model (3 weeks)	↓ intestinal permeability and oxidative stress; strongest GABAergic effect among tested strains	↑ GABAergic receptor expression; strongest effect vs. L. bulgaricus and PROTEXIN^®^	GABAergic	[152]
*Lactobacillus helveticus*	Sprague-Dawley rats; acute brain injury (15 days)	Neuroprotective effects confirmed via bicuculline antagonism	↑ GABA_A_ α2, β2, γ2 subunits; ↑ GABA_B_ in hippocampus and striatum	GABAergic (GABA_A_ α2, β2, γ2; GABA_B)_	[155]
*Limosilactobacillus reuteri* (standalone)	Cntnap2 heterozygous deletion mice; autism model (4 weeks)	Restoration of GABAergic alterations in ventral hippocampus	↑ GABA_A_ γ subunit expression in ventral hippocampus (restored from deficit)	GABAergic (GABA_A_ γ)	[157]
*Enterococcus faecalis* EC-12	Male C57BL/6J mice; dietary supplementation (4 weeks)	↓ anxiety-like behaviors; no effect on dopaminergic or serotonergic receptors	↑ Adrb3 (β3-adrenergic) and Avpr1a (vasopressin 1a) receptor expression	Adrenergic (Adrb3); Vasopressinergic (Avpr1a)	[161]
*Lacticaseibacillus rhamnosus* JB-1 + *Limosilactobacillus reuteri* 6475	Mice (28 days)	↓ JAK2 expression; modulation of serotonergic genes and BDNF	Combined interaction of neurotrophic and immunological pathways	JAK/STAT; serotonergic; BDNF/TrkB	[163]
*B. bifidum* novaBBF7 + *B. longum* novaBLG2 + *L. paracasei* TJB8	In vitro: neuronal (PC12) and monocytic (THP-1) cells	↑ BDNF expression; ↑ p-Akt levels	Activation of BDNF/TrkB–PI3K/Akt pathway; promotion of neuronal survival	PI3K/Akt; BDNF/TrkB	[19]
*Bactolac* (*L. plantarum* NBIMCC 8767 + *S. thermophilus* NBIMCC 8258)	Wistar rats; chronic stress/depression model (8 weeks)	↓ depressive behaviors; modulation via BDNF and NLRP3	↓ 5-HT_1A_, DRD1, ADRA-2A, CNR1, NR3C2 receptor expression	Serotonergic (5-HT_1A_); Dopaminergic (DRD1); Adrenergic (ADRA-2A); Endocannabinoid (CNR1)	[160]
*Bifidobacterium* strains (gut bifidobacteria)	Healthy adult rats; GF mice (multiple studies)	↑ hippocampal plasticity and cognitive behavior; normalization of neurogenesis in GF; ↑ DCX+ cells in dentate gyrus	↑ LTP; ↑ synapse formation; ↑ BDNF; proper refinement of developing synaptic network	BDNF/TrkB; neurotrophic factors	[112,113,114]

Abbreviations: DCX, doublecortin; NMDAR, N-methyl-D-aspartate receptor; GABA_A_/_B_, gamma-aminobutyric acid receptor types A and B; PI3K/Akt, phosphoinositide 3-kinase/protein kinase B. ↑ = increase; ↓ = decrease.

## Data Availability

No new data were created or analyzed in this study. Data sharing is not applicable to this article.

## Abbreviations

The following abbreviations are used in this manuscript:

MGB	Microbiota–gut–brain
SCFAs	Short-chain fatty acids
BDNF	Brain-derived neurotrophic factor
GF	Germ-Free
MDD	Major depressive disorder
AHN	Adult hippocampal neurogenesis
DG	Dentate gyrus
HPA	Hypothalamic–pituitary–adrenal
mPFC	Medial prefrontal cortex
GABA	Gamma-aminobutyric acid
fMRI	Functional magnetic resonance imaging
5-HT	Serotonin
DA	Dopamine
NE	Norepinephrine
ENS	Enteric nervous system
MRIs	Structural magnetic resonance imaging
DTI	Diffusion tensor imaging
EEG	Electroencephalogram
BBB	Blood–brain barrier
ASD	Autism spectrum disorders
ADHD	Attention deficit hyperactivity disorder
EECs	Enteroendocrine cells
PAMPs	Pathogen-associated molecular patterns
CNS	Central nervous system
NTS	Nucleus of the solitary tract
DMV	Dorsal motor nucleus of the vagus nerve
TNF	Tumor necrosis factor
LPS	Lipopolysaccharide
TLRs	Toll-like receptors
IL-1β	Interleukin-1 beta
IL-6	Interleukin-6
TNF-α	Tumor Necrosis Factor-alpha
IFN-γ	Interferon-gamma
PYY	Peptide YY
GLP-1	Glucagon-like peptide-1
NPCs	Neural progenitor cells
HDACs	Histone deacetylases
LTP/LTD	Long-term potentiation/Long-term depression
PSD-95	Postsynaptic density protein 95
NGF	Nerve growth factor
GLIN	Glial cell lineage-derived neurotrophic factor
JAK/STAT	Janus Kinase/Signal Transducer and Activator of Transcription
NMDA	N-Methyl-D-Aspartate
CUMS	Chronic unpredictable mild stress
NMDAR2B	N–methyl–D–aspartate Receptor 2B
NMDAR1	N–methyl–D–aspartate Receptor 1
NMDA2A	N–methyl–D–aspartate Receptor 2A
mRNA	messenger RNA
GAD	Glutamate decarboxylase
5-HT_1A_	5-Hydroxytryptamine 1A receptor
DRD1	Dopamine receptor D1
ADRA-_2A_	Alpha-2A adrenergic receptor
NR3C2	Mineralocorticoid receptor
CNR1	Cannabinoid receptor type 1
Adrb3	β3-adrenergic receptor
Avpr1a	Vasopressin 1a receptor
Drd5	Dopamine Receptor D5
Htr2b	5-hydroxytryptamine receptor 2B
SERT	5-HT transporter
SSRIs	Selective serotonin reuptake inhibitors
THP-1	Human monocytic leukemia cell line
IBS	Irritable bowel syndrome
miRNAs	microRNAs
NPY	Neuropeptide Y
RCTs	Randomized controlled trials
CSF	Cerebrospinal fluid
n-3 PUFA	Omega-3 polyunsaturated fatty acids
EPA	Eicosapentaenoic acid
DHA	Docosahexaenoic acid
VEGF-A	Vascular endothelial growth factor-A
DCX	Doublecortin
GABA_A_/_B_	Gamma-aminobutyric acid receptor types A and B
PI3K/Akt	Phosphoinositide 3-kinase/protein kinase B
RCT	Randomized controlled trial
OB	Oral probiotic
iPSC	Induced pluripotent stem cells
FMT	Fecal microbiota transplantation
